# Modeling and evaluation of BVOCs-mediated plant-to-plant stress signaling: a molecular communication perspective

**DOI:** 10.3389/fpls.2026.1795657

**Published:** 2026-05-18

**Authors:** Yusheng Sun, Hang Zhang, Pengfei Zhang, Ping Zhou, Pengfei Lu

**Affiliations:** College of Information Science and Technology, Shihezi University, Shihezi, China

**Keywords:** biogenic volatile organic compounds, channel modeling, molecular communication, plant communication, stress, testbed

## Abstract

During plant growth and development, diverse environmental stresses can disrupt normal physiological activities, often leading to growth inhibition or death. Plants release biogenic volatile organic compounds as key mediators to communicate these stress signals to neighboring individuals. However, complex environmental factors—such as meteorological conditions and atmospheric turbulence—markedly affect the atmospheric transport of these compounds. To address the limitations of existing studies, we model the atmospheric dispersion of a single biogenic volatile organic compound species as a single-input single-output molecular communication system, focusing on its instantaneous release under specific wind-speed constraints. By jointly considering plant height, turbulent diffusion, wind speed, and photochemical oxidative degradation, we derive an analytical expression for the received concentration. In this framework, the emitting plant serves as the transmitter, the target plant as the receiver, and the air as the channel. Finally, using numerical simulations and a molecular communication test platform, we systematically conduct transmission performance analysis and evaluate the model through parameter fitting. This research presents a rigorous mathematical framework for analyzing inter-plant signaling, laying the theoretical groundwork for intelligent agricultural applications, such as biogenic volatile organic compounds mediated environmental sensing and precision regulation.

## 1 Introduction

In plants, environmental stresses can markedly disrupt physiological and metabolic processes, resulting in growth inhibition, abnormal development, or even death (Ranieri et al., 2021). Statistics from the Food and Agriculture Organization of the United Nations indicate that stress-related yield losses account for approximately 30% of global crop production each year (Verma et al., 2025). Because plants cannot escape unfavorable conditions through migration, they must balance physiological homeostasis with growth and development under resource constraints when exposed to stress. Over evolutionary time, plants have developed the capacity to convey stress information by emitting biogenic volatile organic compounds (BVOCs) (Satake et al., 2024; Ninkovic et al., 2021), enabling rapid dissemination of stress signals within plant communities and triggering coordinated defense responses (Hirose and Satake, 2024). Understanding how these airborne chemical signals are generated, transported, and perceived is therefore essential for advancing both fundamental plant science and agricultural applications.

Substantial progress has been made in elucidating the biological mechanisms underlying BVOCs-mediated communication. Traditional studies of inter-plant information exchange have primarily relied on observational and biochemical methodologies to characterize signal compositions and transmission pathways. Advanced analytical techniques such as HS-SPME-GC-QTOF-MS have enabled comprehensive profiling of BVOCs from plant tissues, revealing distinct emission patterns across different species and environmental conditions (Fuente-Ballesteros et al., 2025). The latest developments in PTR-TOF-MS offer the capability for high-resolution, real-time monitoring of BVOCs emission dynamics. Thanks to its adaptable analytical workflow, the technology effectively handles the complex volatile mixtures encountered in studies with multiple replicates and treatment factors (Fontez et al., 2025). With respect to transmission mechanisms, rhizosphere microorganisms have been shown to produce specific signaling molecules that modulate plant root architecture and subsequently influence above-ground growth (Li et al., 2024). At the plant–plant interaction level, volatile-mediated bidirectional communication has been demonstrated, where receiver plants emit feedback signals (e.g., squalene) that enhance stress tolerance in emitter plants through the squalene-castasterone-BES1/BZR1-CBF5 signaling pathway (Jin et al., 2025). Complementary evidence shows that specific volatile cues trigger stomatal responses and activate downstream gene expression networks in receiver plants, with communication specificity modulated by the genetic relatedness between interacting individuals (Ito et al., 2025). The ecological relevance of such communication has been validated in complex agricultural systems, where aphid-infested cucumber plants induce oxidative stress responses and reshape rhizosphere microbial communities in neighboring plants, consequently enhancing their natural resistance to herbivores (Liu et al., 2024). Field experiments using transgenic silver birch with enhanced isoprene emission further demonstrated that airborne isoprene can induce systemic acquired resistance in neighboring Arabidopsis against Pseudomonas syringae through LLP1-dependent pathways (Zhu et al., 2025). Collectively, these advances have elucidated the molecular basis and ecological significance of plants communication. However, the above studies focus predominantly on the biochemical generation and perception of signals at the transmitter and receiver ends, while the physical processes governing how BVOCs are transported through the atmosphere from one plant to another remain largely unexplored.

A growing body of evidence suggests that environmental and ecological factors critically shape BVOCs transport, yet current understanding remains largely qualitative. Atmospheric CO_2_ concentrations regulate emission rates (Stringari et al., 2024), while interactions among soil nutrients, warming, and shading alter both the intensity and spectral composition of transmitted signals (Ndah et al., 2022). Spatially explicit evolutionary models have suggested that narrow signaling ranges favor the evolution of BVOCs mediated communication (Hirose and Satake, 2024), while field observations show that proximity to BVOCs source areas correlates with enhanced salicylic acid accumulation in downwind receiver trees (Hagiwara et al., 2024). Notably, in natural plant communities, neighboring plants situated between the emitter and receiver can physically obstruct airflow and absorb volatile molecules, thereby attenuating signal concentration at the receiver. Such shielding and absorptive effects have been documented in studies on windbreak-induced turbulence modification (Bannister et al., 2022). Despite these insights, current research lacks a unified mechanistic framework that integrates physical transport, environmental modulation, and the shielding effects of neighboring vegetation to quantitatively predict inter-plant BVOCs propagation.

To address this limitation and bridge the gap between conceptual models and quantitative analysis, recent advances in molecular communication (MC) theory offer a promising approach. Emerging from the convergence of information science and life sciences, MC provides a unified quantitative framework for characterizing information transfer in biological systems (Huang et al., 2021; Kuran et al., 2020). Unlike traditional biochemical models, which focus on individual molecular interactions, MC adopts a systems level perspective to analyze end-to-end communication performance. Grounded in information theory and communication principles, MC maps processes such as the generation, release, diffusion, reaction, and detection of molecular signals onto the transmitter, channel, and receiver of a communication system, thereby enabling evaluation of key performance metrics including transmission efficiency, interaction range, and error characteristics (Chouhan and Alouini, 2023; Asghar et al., 2022). This framework has been applied to diverse biological contexts, from intracellular calcium signaling and quorum sensing in bacteria to pheromone-based communication in insects (Bi et al., 2021). Recent applications to plant systems have incorporated BVOCs propagation into MC models (Kilic and Akan, 2025) and proposed modulation schemes for characterizing information transfer and competitive interference among multiple plants (Aktas et al., 2024). These studies have explored fundamental channel characteristics such as impulse response, signal-to-noise ratio, and mutual information under simplified diffusion scenarios. Nevertheless, existing MC-based models have not yet coupled channel dynamics with the physical and ecological processes governing BVOCs transport in natural environments.

Accordingly, this study develops and validates a modeling framework for BVOCs-mediated information transfer among plants in natural environments under the molecular communication paradigm. The main contributions are as follows: 1. Multi-factor BVOCs propagation model: We develop a parameterized model that incorporates emitter–receiver height differences, turbulence, wind variability, and photochemical oxidation to predict atmospheric BVOCs transport and concentration fields under realistic conditions. 2. Interfering-plant modeling: We introduce a mechanistic representation of neighboring plants as physical and absorptive obstacles within the communication channel, quantifying their signal attenuation effects on the received concentration. 3. Numerical simulation and experimental validation: We validate the proposed model through numerical simulations and controlled experiments on a molecular communication testbed, assessing key transport parameters and environmental drivers.

## 2 Materials and methods

### 2.1 System description

**Figure 1:**
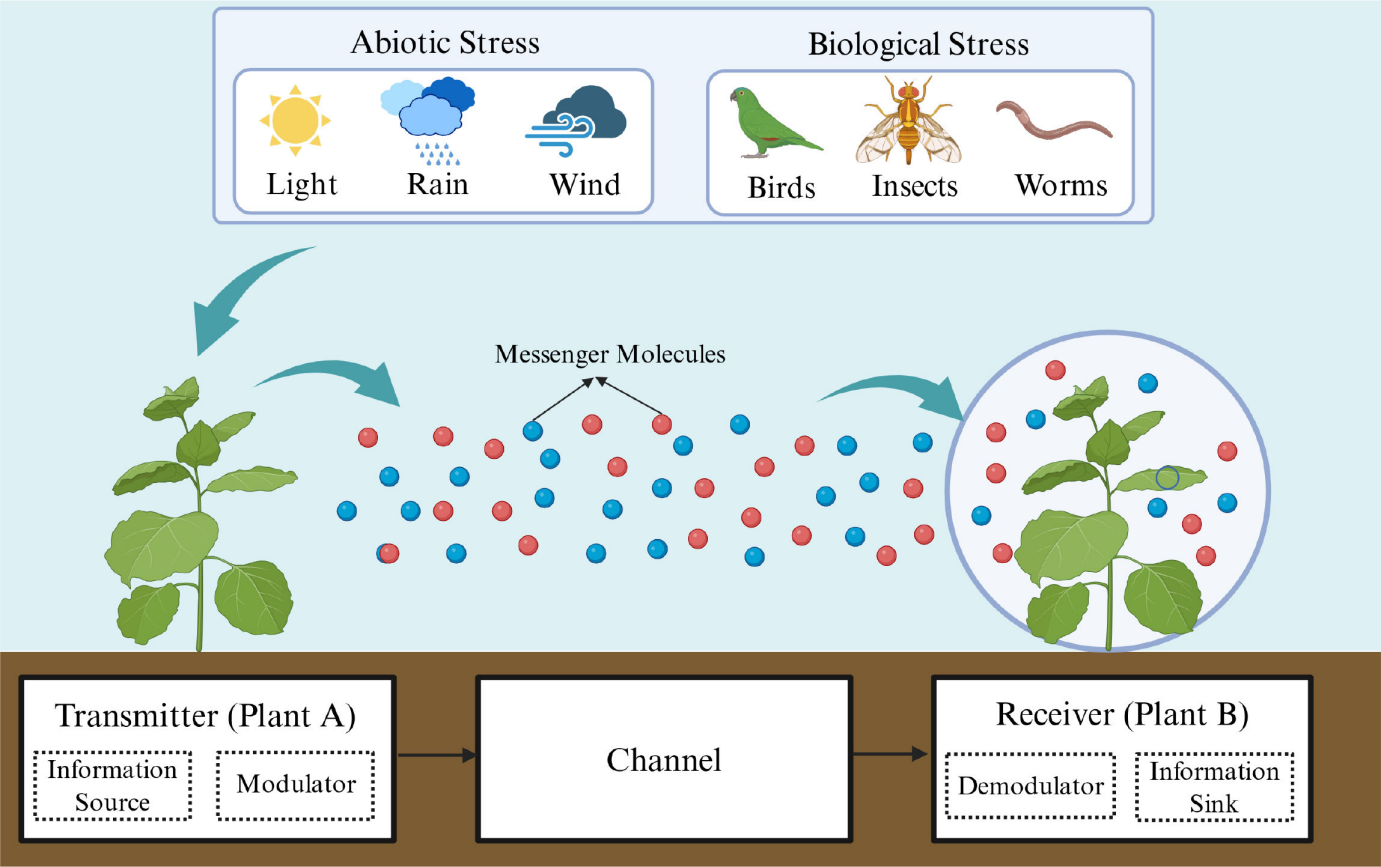
MC framework for inter-plant signaling. The plant on the left (Plant A) acts as the transmitter and, upon stress exposure, releases BVOCs to convey stress information to the plant on the right (Plant B). In this process, Plant A encodes the stress into corresponding molecular signals, while Plant B decodes the received signals to identify the specific stress type. The air medium serves as the transmission channel. Adapted from (Sun et al., 2025).

To quantitatively elucidate the transmission of stress-related information among plants, this section develops a single-input single-output (SISO) BVOCs propagation model within the molecular communication framework, integrating multiple environmental influencing factors (Yaylali et al., 2023). Unlike traditional studies of plant signaling, which primarily focus on physiological responses or molecular recognition, this work models the airborne propagation of stress signals quantitatively at the system level, with an emphasis on the diffusion and transport characteristics of BVOCs in the environment. The overall system architecture is shown in **Figure 1**, where Plant A acts as the transmitter, the air medium serves as the channel, and Plant B functions as the receiver. For mathematical tractability and to rigorously isolate the physical transport mechanisms, we impose three well-defined assumptions:(1) The analysis is restricted to biotic and abiotic stress types known to induce robust BVOCs emissions. (2) Each stress type is associated with the release of a single dominant indicator compound, and secondary chemical interactions among complex blend components are neglected. While natural plant emissions constitute realistic diverse BVOCs mixtures, utilizing a primary tracer is a standard and necessary abstraction in foundational atmospheric transport and MC models. This simplification prevents the physical transport equations from being mathematically underdetermined by highly non-linear atmospheric cross-chemistry, thereby allowing for a clear, decoupled evaluation of the spatial-temporal attenuation of the communication channel. (3) The biological layer is treated conceptually rather than mechanistically. Highly complex biological processes, such as continuous emission dynamics, stomatal regulation, and receptor biochemistry, are abstracted to focus purely on the atmospheric channel. Consequently, the boundary conditions are explicitly defined: the emission at the transmitter is modeled as a simplified binary source term formulated as a time-varying mass flux boundary condition, while the uptake at the receiver is defined as a concentration-dependent mass transfer boundary. This clear definition avoids overstating the biological integration while maintaining its focus on the environmental propagation dynamics rather than internal plant metabolism.

### 2.2 Modulation and demodulation mechanism

**Figure 2:**
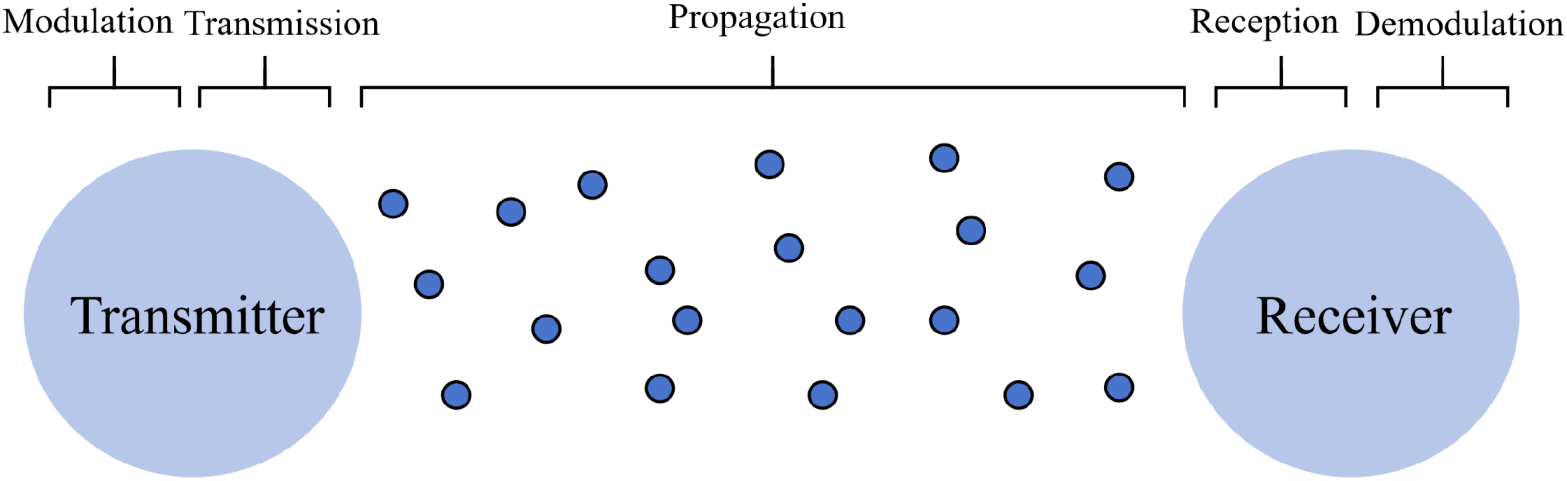
Molecular communication system framework.

MC theory abstracts information transfer into a molecule-mediated communication system, providing a powerful framework for quantitatively studying biological signaling interactions. As shown in **Figure 2**, the system consists of a transmitter, a receiver, a propagation channel, and a signal modulation/demodulation module (Brand et al., 2022). The key idea is that the transmitter modulates information by controlling the release of information-carrying molecules, while the receiver demodulates the signal by sensing the spatiotemporal dynamics of molecular concentration or flux.

Within this framework, stress-induced BVOCs emission from the transmitter plant serves as the information source, with the released molecules propagating through the atmospheric medium to the receiver plant. Environmental factors—such as wind velocity, temperature gradients, and humidity fluctuations—together with ambient noise, introduce perturbations that degrade signal integrity and may lead to decoding errors. The receiver plant identifies the specific stress type based on the quantified quantity of BVOCs uptake.

To mathematically characterize this communication process, we formulate a quantitative model of BVOCs release and propagation. It should be noted that real plant stress signaling is inherently continuous and dynamic, and the assumption of binary stress encoding does not fully capture the complexity of most natural ecological interactions. Nevertheless, as a necessary first step to establish a tractable molecular communication framework, we adopt an on–off keying (OOK) modulation scheme as a mathematically foundational baseline. While this abstraction discretizes continuous biological events into binary logic, it serves as a valid extreme-case approximation to model discrete, acute stress events (e.g., sudden severe mechanical damage). Under this simplified scheme, BVOCs are released under acute stress stimulation (corresponding to bit “1”), whereas no release occurs in the absence of stimulation (corresponding to bit “0”). Accordingly, the signal manifests in the time domain as an on–off sequence, which can be mathematically expressed as Equation 1:

(1) $$S=\{\begin{array}{ll} m, & \text{if bit}=1,\\ 0, & \text{if bit}=0 \end{array},$$

where *S* denotes the source term and *m* represents the total instantaneous mass of BVOCs released as a discrete bolus at the beginning of a signaling interval. When the bit is “1”, the plant emits BVOCs, generating a valid signal; when the bit is “0”, the plant remains in a resting state and no emission occurs.

Subsequently, signal demodulation at the receiver plant employs a detection threshold denoted as *m*_th_. The selection of this threshold is biologically grounded, representing the sensitivity limit of the receiver’s olfactory receptors and defining the minimum accumulated quantity required to trigger downstream physiological responses while filtering out ambient environmental background noise. If the received quantity of BVOCs, denoted as *m*_recv_, exceeds or equals this threshold, the transmitter is deemed in the “on” state (*bit* = 1), indicating stress signal detection; conversely, if the received quantity is below the threshold, it is classified as the “off” state (*bit* = 0), indicating no stress signal is detected. This decision rule can be expressed as follows Equation 2:

(2) $$bit=\{\begin{array}{ll} 0, & \text{if }m_{\text{recv}}<m_{\text{th}},\\ 1, & \text{if }m_{\text{recv}}\geq m_{\text{th}} \end{array}.$$

### 2.3 Channel mode

#### 2.3.1 Turbulent mixing and convective transport effects

Upon exposure to stress stimuli, the transmitter plant releases internally synthesized BVOCs into the atmosphere. The spatiotemporal transport of these molecules is governed by environmental conditions and diffusion dynamics. To quantitatively characterize the BVOCs distribution at the receiver, we establish a concentration field model describing the BVOCs concentration at any location (*x,y,z*) and time *t*. We consider propagation dominated by turbulent diffusion and mean-wind advection. Assuming the transmitter is located at [0, 0*, H*] and instantaneously releases a quantity of BVOCs at *t* = 0, the corresponding source term is formulated as Equation 3:

(3) S=m δ(x)δ(y)δ(z−H)δ(t),

where *δ*(*x*) denotes the Dirac delta function, and *H* is the height of the transmitter leaf above the ground. Then, according to the law of mass conservation, the time derivative of the BVOCs concentration *C*(*x, y, z, t*) at an arbitrary location in space can be written as Equation 4:

(4) $$\frac{\partial C(x,y,z,t)}{\partial t}+\nabla \cdot \vec{J}=S,$$

where the vector function $\vec{J}$ represents the mass flux of the infochemical induced by advection and diffusion, and can be expressed as Equation 5:

(5) $$\vec{J}={\vec{J}}_{A}+{\vec{J}}_{D},$$

where ${\vec{J}}_{A}$ denotes the convective flux induced by the wind field and can be expressed as Equation 6:

(6) $${\vec{J}}_{A}=\vec{v}\;C(x,y,z,t),$$

where $\vec{v}$ is the mean flow velocity vector. In this section, we consider only the advective wind component in the < direction to obtain a closed-form expression for the concentration distribution; thus $\vec{v}=[u,0,0]$, where *u* denotes the wind speed and is assumed constant. Therefore, $\nabla \cdot {\vec{J}}_{A}$ can be expressed as Equation 7:

(7) $$\nabla \cdot {\vec{J}}_{A}=\nabla \cdot (\vec{v}\;C(x,y,z,t))=u\;\frac{\partial C(x,y,z,t)}{\partial x},$$

where ${\vec{J}}_{D}$ represents the diffusion induced by turbulent eddy motions in the atmosphere. According to Fick’s second law, the diffusive flux varies linearly with the concentration gradient; therefore, ${\vec{J}}_{D}$ can be expressed as Equation 8:

(8) $${\vec{J}}_{D}=-K\nabla C(x,y,z,t),$$

Subsequently, to obtain a closed-form analytical solution, we make the following assumptions: (1) diffusion is isotropic and the eddy diffusivity depends only on the downwind distance *x*, i.e., $K_{x}(x)=\;K_{y}(x)=K_{z}(x)\;:=K(x)$. (2) The flow field satisfies the incompressibility condition, i.e., the air density does not vary significantly with time or space. (3) Owing to the relatively high wind speed, advective transport dominates in the x-direction, and the diffusion term in the x-direction is negligible compared with the advection term, thus Equation 9.

(9) $$\frac{\partial }{\partial x}(K(x)\;\frac{\partial C(x,y,z,t)}{\partial x})=0,$$

These assumptions, which are applicable to turbulent diffusion problems dominated by the wind field, have been adopted in the literature (Kilic and Akan, 2025). Substituting Equations 7 and Equations 8 into the mass-conservation Equations 4 and applying the above assumptions yield the governing equation Equation 10:

(10) $$\frac{\partial C(x,y,z,t)}{\partial t}+u\;\frac{\partial C(x,y,z,t)}{\partial x}-K(x)(\frac{\partial^{2}C(x,y,z,t)}{\partial y^{2}}+\frac{\partial^{2}C(x,y,z,t)}{\partial z^{2}})=m\;\delta (x)\delta (y)\delta (z-H)\delta (t),$$

where $\frac{\partial C(x,y,z,t)}{\partial x}$ denotes the wind-driven transport effect along the x-axis, $K(x)\frac{\partial^{2}C(x,y,z,t)}{\partial y^{2}}$ and $K(x)\frac{\partial^{2}C(x,y,z,t)}{\partial z^{2}}$ represent the turbulent diffusion in the y and z directions, respectively. To solve Equations 10, the boundary conditions must be specified. In this study, we adopt the following boundary conditions: $C(0,y,z,t)=\frac{m}{u}\;\delta (y)\delta (z-H)\delta (t),C(\infty ,y,z,t)=0,C(x,\infty ,z,t)=0,C(x,y,\infty ,t)=0,K(x)\frac{\partial C(x,y,0,t)}{\partial z}=0$. Subsequently, by applying the Stakgold theorem, Equations 10 can be recast as Equation 11:

(11) $$\frac{\partial C(x,y,z,t)}{\partial t}+u\;\frac{\partial C(x,y,z,t)}{\partial x}-K(x)(\frac{\partial^{2}C(x,y,z,t)}{\partial y^{2}}+\frac{\partial^{2}C(x,y,z,t)}{\partial z^{2}})=0.$$

However, in practice, the eddy diffusivity *K*(*x*) is difficult to determine accurately. To eliminate the explicit appearance of *K*(*x*) in the equation, we introduce a new spatial variable *r*, defined as Equation 12:

(12) $$r=\frac{1}{u}\int_{0}^{x}K(\delta )\;d\delta ,$$

Physically, the variable *r* has the dimension of area and represents the cumulative spatial variance (or the accumulated dispersion area) of the BVOCs plume advected from the source to distance x. This transformation maps the physically heterogeneous domain into a homogeneous diffusion space, thereby absorbing the spatially varying K(x) into the new coordinate system. Accordingly, we obtain $\frac{dr}{dx}=\frac{K(x)}{u}$ and $\frac{\partial C(x,y,z,t)}{\partial x}=\frac{K(x)}{u}\;\frac{\partial C(x(r),y,z,t)}{\partial r}$. Substituting the above transformation into Equations 11 and applying the chain rule yields Equation 13:

(13) $$\frac{\partial C(x(r),y,z,t)}{\partial t}+K(x)\frac{\partial C(x(r),y,z,t)}{\partial r}-K(x)(\frac{\partial^{2}C(x(r),y,z,t)}{\partial y^{2}}+\frac{\partial^{2}C(x(r),y,z,t)}{\partial z^{2}})=0,$$

Dividing both sides by K(x) yields Equation 14:

(14) $$\frac{1}{K(x)}\frac{\partial C(x(r),y,z,t)}{\partial t}+\frac{\partial C(x(r),y,z,t)}{\partial r}=\frac{\partial^{2}C(x(r),y,z,t)}{\partial y^{2}}+\frac{\partial^{2}C(x(r),y,z,t)}{\partial z^{2}},$$

Subsequently, in the new coordinate system, we define C"(r,y,z,t)=C(x(r),y,z,t). Considering a typical atmospheric dispersion environment, for advection-dominated transport along the main stream direction, the adjustment rate of the concentration field in the streamwise direction is much faster than the expansion rate in the transverse direction. Therefore, under these circumstances, the concentration field quickly establishes a quasi-steady distribution along the wind direction, and the temporal variation of concentration is negligible compared to the spatial gradient terms (Osnes et al., 2023). Consequently, the coefficient $\frac{1}{K(x)}$ preceding the time derivative term can be absorbed through non-dimensionalization (Singh and Singh, 2022), yielding Equation 15:

(15) $$\frac{\partial C^{\prime }(r,y,z,t)}{\partial t}+\frac{\partial C^{\prime }(r,y,z,t)}{\partial r}=\frac{\partial^{2}C^{\prime }(r,y,z,t)}{\partial y^{2}}+\frac{\partial^{2}C^{\prime }(r,y,z,t)}{\partial z^{2}},$$

The boundary conditions remain as specified in Equations 10 . Since Equations 15 is a linear partial differential equation with constant coefficients, the solution can be obtained by separation of variables. Specifically, we seek a solution of the following product form Equation 16:

(16) $$C^{\prime }(r,y,z,t)=\frac{m}{u}\;Y(r,y)Z(r,z)L(r,t),$$

This form allows the spatial and temporal components to be separated, enabling each part to be treated independently. Consequently, we obtain three reduced problems for the functions *Y* (*r,y*), *Z*(*r,z*), and *L*(*r,t*), respectively, as shown below Equation 17–19:

(17) $$\frac{\partial Y(r,y)}{\partial r}=\frac{\partial^{2}Y(r,y)}{\partial y^{2}},$$

(18) $$\frac{\partial Z(r,z)}{\partial r}=\frac{\partial^{2}Z(r,z)}{\partial z^{2}},$$

(19) $$\frac{\partial L(r,t)}{\partial t}=-\frac{\partial L(r,t)}{\partial r},$$

The boundary conditions for Equations 17 are: Y(0,y)=δ(y),Y(∞,y)=0,Y(r,∞)=0; those for Equations 18 are: $Z(0,z)=\delta (z-H),Z(\infty ,z)=0,Z(r,\infty )=0,\frac{\partial Z(r,0)}{\partial z}=0$; and those for Equations 19 are: $L(0,t)=\delta (t),L(\infty ,t)=0,L(r,\infty )=0,\frac{\partial L(r,0)}{\partial t}=0$. To solve these equations, we employ the Fourier transform to reduce each partial differential equation to an ordinary differential equation, thereby simplifying the solution process.

To solve Equation 17, we apply the Fourier transform with respect to *y*, defined as Equation 20:

(20) $$\hat{Y}(r,k)=F\{Y(r,y)\}=\int_{-\infty }^{+\infty }e^{-iky}Y(r,y)\;dy,$$

Applying this to Equation 17, the left-hand side becomes Equation 21:

(21) $$F\{\frac{\partial Y}{\partial r}\}=\frac{\partial \hat{Y}(r,k)}{\partial r},$$

For the right-hand side, we apply the differentiation property of the Fourier transform, $F\{\frac{\partial^{2}Y}{\partial y^{2}}\}=-k^{2}\hat{Y}$, to obtain Equation 22:

(22) $$\frac{\partial \hat{Y}(r,k)}{\partial r}=-k^{2}\hat{Y}(r,k),$$

This is a first-order linear ordinary differential equation with respect to *r*, which can be solved by separation of variables, yielding Equation 23:

(23) $$\hat{Y}(r,k)=A(k)e^{-k^{2}r},$$

Here, A(k) is an unknown function to be determined by the initial condition. From Y(0,y)=δ(y), taking the Fourier transform yields $\hat{Y}(0,k)=F\{\delta (y)\}=1$. Setting r=0 in Equation 23 gives $\hat{Y}(0,k)=A(k)$. Therefore, A(k)=1, and the solution becomes Equation 24:

(24) $$\hat{Y}(r,k)=e^{-k^{2}r},$$

Next, we apply the inverse Fourier transform to obtain *Y* (*r,y*) Equation 25:

(25) $$Y(r,y)=F^{-1}\{\hat{Y}(r,k)\}=\frac{1}{2\pi }\int_{-\infty }^{+\infty }e^{-k^{2}r+iky}\text{ d}k,$$

To evaluate this integral, we use the standard Gaussian integral formula $\int_{-\infty }^{\infty }e^{-ak^{2}+bk}dk=\sqrt{\frac{\pi }{a}}\text{exp }(\frac{b^{2}}{4a})$ with *a* = *r* and *b* = *iy*, we obtain Equation 26:

(26) $$Y(r,y)=\frac{1}{2\pi }\sqrt{\frac{\pi }{r}}\text{exp }(-\frac{y^{2}}{4r})=\frac{1}{2\sqrt{\pi r}}\text{exp }(-\frac{y^{2}}{4r}).$$

Subsequently, applying the same procedure to Equation 18, Equation 19 yields Equations 27, 28:

(27) $$Z(r,z)=\frac{1}{2\sqrt{\pi r}}(\text{exp }(-\frac{{\left(z-H\right)}^{2}}{4r})+\text{exp }(-\frac{{\left(z+H\right)}^{2}}{4r})),$$

(28) $$L(r,t)=\frac{u}{2\sqrt{\pi r}}\text{exp }(-\frac{{\left(x-ut\right)}^{2}}{4r}),$$

Finally, combining Equation 16, Equation 26, Equation 27 and Equations 28, we obtain Equation 29:

(29) $$C(x,y,z,t)=\frac{m}{{\left(4\pi r\right)}^{3/2}}\text{exp}(-\frac{{\left(x-ut\right)}^{2}-y^{2}}{4r})[\text{exp}(-\frac{{\left(z-H\right)}^{2}}{4r})+\text{exp}(-\frac{{\left(z+H\right)}^{2}}{4r})],$$

However, in natural environments, the eddy diffusivities along the *y* and *z* axes are generally unequal. Therefore, Equation 29 can be rewritten as Equation 30:

(30) $$C(x,y,z,t)=\frac{m}{{\left(4\pi \sqrt{r_{y}r_{z}}\right)}^{3/2}}\text{exp}(-\frac{{\left(x-ut\right)}^{2}}{4\sqrt{r_{y}r_{z}}})\text{exp}(-\frac{y^{2}}{4r_{y}})[\text{exp}(-\frac{{\left(z-H\right)}^{2}}{4r_{z}})+\text{exp}(-\frac{{\left(z+H\right)}^{2}}{4r_{z}})].$$

where, $r_{y}(x)=\frac{1}{u}\int_{0}^{x}K_{y}(\delta )\;d\delta ,r_{z}(x)=\frac{1}{u}\int_{0}^{x}K_{z}(\delta )\;d\delta$.

Eddy diffusivities vary with atmospheric stability, which depends on meteorological conditions. Because an explicit analytical relation between eddy diffusivity and transport distance is difficult to establish, we adopt the Pasquill stability classification scheme (Tachibana et al., 2025), which uses wind speed and the standard deviations of horizontal and vertical wind fluctuations to characterize atmospheric dispersion. This scheme divides atmospheric stability into six classes (A–F) based on meteorological factors (e.g., temperature gradient, wind speed, and turbulence intensity), each associated with distinct eddy-diffusion properties that govern the transport of BVOCs. In this study, we adopt stable (Class F) atmospheric conditions as a representative scenario to derive an analytically tractable dispersion model. We acknowledge that Class F corresponds primarily to stable, nighttime-like conditions and may not fully capture the turbulent mixing characteristic of unstable daytime environments, where BVOCs signaling is often most active. Extending the framework to other stability classes (Class A–C for daytime convective conditions) constitutes an important direction for future work. Under Class F conditions, we model BVOCs dispersion as follows Equations 31, 32:

(31) $$r_{y}(x)=\frac{0.08x^{2}}{1+0.0001x},$$

(32) $$r_{z}(x)=\frac{0.000128x^{2}}{{\left(1+0.0003x\right)}^{2}}.$$

#### 2.3.2 Buoyancy and gravity effects

In the previous section, BVOCs transport was modeled via turbulent diffusion and wind-field advection. In realistic atmospheric contexts, BVOCs are trace gaseous molecules, and their transport is overwhelmingly dominated by turbulent mixing, while true gravitational settling of individual gas molecules is practically negligible. However, to theoretically explore the potential upper bound of density-driven vertical drift during the initial emission phase (where BVOCs might briefly act as a localized, higher-concentration gas puff), we introduce an idealized mathematical abstraction. In this simplified framework, the locally emitted BVOCs are conceptually treated as a macroscopic parcel with volume *V* and effective density *ρ_b_* to evaluate the maximum possible influence of buoyancy *F_B_* and gravity *F_G_*. The theoretical buoyancy force is given by Equation 33:

(33) $$F_{B}=\rho_{a}Vg=\frac{PM}{RT}Vg,$$

where *ρ_a_* is the ambient air density, *V* is the volume of the idealized BVOCs parcel, and *g* is the gravitational acceleration. Using the ideal-gas equation of state, $\rho_{a}=\frac{PM}{RT}$, where *P* is atmospheric pressure, *M* is the molar mass of air, *R* is the universal gas constant, and *T* is the absolute temperature.

In addition to the upward buoyancy force, the macroscopic parcel is influenced by its theoretical weight. The gravitational force *F_G_* can be expressed as Equation 34:

(34) $$F_{G}=m_{b}g=\rho_{b}Vg,$$

Thus, the net vertical force *F_z_* on this idealized parcel along the *z*-axis is Equation 35:

(35) $$F_{z}=F_{B}-F_{G}=V(\frac{PM}{RT}-\rho_{b})g,$$

In practice, the vertical motion of airborne parcels is heavily dampened by aerodynamic drag, which rapidly leads to a constant terminal velocity rather than continuous acceleration. Nevertheless, to establish a mathematical envelope that captures the maximum theoretical extent of this vertical drift over short emission distances, vertical air resistance is temporarily neglected in this baseline model. By deliberately utilizing this frictionless condition to define the upper boundary of the drift, applying Newton’s second law and integrating the equation of motion yields the upper-bound vertical displacement *s_z_*(*t*) along the *z*-axis Equation 36:

(36) $$s_{z}(t)=\frac{(\frac{PM}{RT}-\rho_{b})g}{2\rho_{b}}t^{2},$$

Therefore, after incorporating this theoretical upper-bound vertical drift, the BVOCs concentration expression from Equations 30 is phenomenologically adjusted as Equation 37:

(37) $$C(x,y,z,t)=\frac{m}{{\left(4\pi \sqrt{r_{y}r_{z}}\right)}^{3/2}}\text{exp}(\frac{-{\left(x-ut\right)}^{2}}{4\sqrt{r_{y}r_{z}}})\text{exp}(\frac{-y^{2}}{4r_{y}})[\text{exp}(\frac{-{\left(z-s_{z}(t)-H\right)}^{2}}{4r_{z}})+\text{exp}(\frac{-{\left(z-s_{z}(t)+H\right)}^{2}}{4r_{z}})].$$

#### 2.3.3 Photochemical oxidation effects

Beyond the influences of wind fields, turbulent diffusion, and buoyancy-gravitational forces, BVOCs experience a suite of complex chemical transformations during atmospheric transport, resulting in concentration attenuation as a function of propagation distance and time. Photochemical oxidation represents one of the dominant chemical loss pathways, wherein BVOCs molecules undergo oxidative reactions with atmospheric species such as molecular oxygen and reactive radicals, yielding oxidized products (Cao et al., 2022). This degradation process progressively reduces the effective BVOCs concentration within the communication channel, thereby attenuating the signal strength detectable by receptor plants.

To quantitatively describe this chemical attenuation mechanism within an analytically tractable communication framework, we adopt a classical chemical kinetics approach. While real-world BVOC oxidation is highly complex and dynamically depends on OH radical concentrations, solar radiation, and oxidant variability, we utilize a macroscopic approximation in this foundational study. Under the assumption that photochemical oxidation follows pseudo-first-order kinetics, the temporal evolution of the BVOCs concentration field *C_l_*(*x, y, z,t)* incorporating photochemical loss can be formulated as (Ezzati et al., 2024) Equation 38:

(38) $$\frac{\text{d}C_{l}(x,y,z,t)}{\text{d}t}=-k_{\text{eff}}C_{l}(x,y,z,t),$$

where *k*_eff_ is the effective oxidation rate constant, which incorporates both the intrinsic reaction rate constant and the oxidant concentration. The effective rate constant *k*_eff_ follows the Arrhenius equation Equation 39:

(39) $$k_{\text{eff}}=A\text{exp }(-\frac{E_{a}}{RT}),$$

where *A* is the pre-exponential factor (frequency factor), *E_a_* is the activation energy, *R* is the gas constant, and *T* is the absolute temperature.

Integrating Equations 38 yields the temporal decay law of the concentration Equation 40:

(40) $$C_{l}(x,y,z,t)=C_{0}\text{exp }(-k_{\text{eff}}t)=C_{0}\text{exp }(-A\text{exp }(-\frac{E_{a}}{RT})t),$$

where *C*_0_ is the concentration distribution from Equations 37 that accounts for buoyancy and gravitational effects. Therefore, the BVOCs concentration expression incorporating photochemical oxidation can be expressed as Equation 41:

(41) $$C_{l}(x,y,z,t)=C(x,y,z,t)\text{exp }(-A\text{exp }(-\frac{E_{a}}{RT})t),$$

Based on Equations 41, we first perform spatial integration over the receptor plant volume, followed by temporal integration from 0 to *τ* seconds. The total quantity *Q* of BVOCs received by the receptor plant over this time period is given by Equation 42:

(42) $$Q=\int_{0}^{\tau }\int_{V}C_{l}(x,y,z,t)\;dV\;dt=\int_{0}^{\tau }\int_{V}C(x,y,z,t)\text{exp }(-A\text{exp }(-\frac{E_{a}}{RT})t)dV\;dt.$$

#### 2.3.4 Effects of interfering plants

In natural ecosystems, interfering plants can influence BVOCs transport via several pathways. Their foliage and canopy structure may absorb, adsorb, or intercept airborne BVOCs, reducing the effective flux. They may also modify flow pathways through spatial obstruction and localized turbulence, and/or emit other volatiles that compete with or mask the target signal, thereby altering BVOCs trajectories and arrival probabilities at the receiver. To capture these effects with manageable complexity, we assume that: (1) only interfering individuals located along the horizontal line of sight connecting the transmitter and the receiver are considered; (2) interfering plants emit no BVOCs and act only as passive sinks; and (3) their effects on the background mean wind and the overall concentration field are negligible.

Under these assumptions, we retain the original governing PDE for the concentration field and, drawing on obstacle-flow and dry-deposition concepts in atmospheric dispersion theory [19], represent the net effect of interfering plants at the path scale. Interfering plants act as passive sinks through stomatal uptake and surface deposition, and their canopy geometry partially impedes transport by altering local concentration gradients. For a fixed transmitter–receiver distance under a given prevailing wind, this combined uptake–blocking effect is approximated as a uniform scaling of the concentration field without interference. We therefore introduce a multiplicative attenuation factor *I*(*α,β*), where *α* is the ratio of the interfering-plant radius to the receptor-plant radius and quantifies the effective blocking area, and *β* is the ratio of the interferer to receiver distance to the total separation and reflects interception efficiency across dispersion stages.

By introducing these two geometric parameters, we can parameterize and adjust the concentration field given by Equations 41. Accordingly, after accounting for interfering plants, the BVOCs concentration at the receiver plant, *C_r_*(*x, y, z, t, α, β*), can be expressed as Equation 43:

(43) $$C_{r}(x,y,z,t,\alpha ,\beta )=I(\alpha ,\beta )C_{l}(x,y,z,t),$$

Similarly, in the presence of interfering plants, the total quantity of BVOCs received by the receptor plant over a given time interval *τ* is denoted by *Q_I_* and can be written as Equation 44:

(44) $$Q_{I}=\int_{0}^{\tau }({∭}_{V}C_{r}(x,y,z,t,\alpha ,\beta )\text{ d}V)\text{d}t=I(\alpha ,\beta )\int_{0}^{\tau }({∭}_{V}C_{l}(x,y,z,t)\text{ d}V)\text{d}t.$$

It should be noted that the attenuation factor *I*(*α,β*) is not a local spatially varying quantity. Rather, it is an equivalent path-scale parameter introduced to characterize the overall adjustment to the cumulative BVOCs flux received under a prescribed spatial configuration of interfering plants. Consequently, *I*(*α,β*) does not explicitly depend on the propagation coordinate *x*; instead, it is determined by the geometric attributes of the interfering plants and their relative positions along the propagation path.

**Figure 3:**
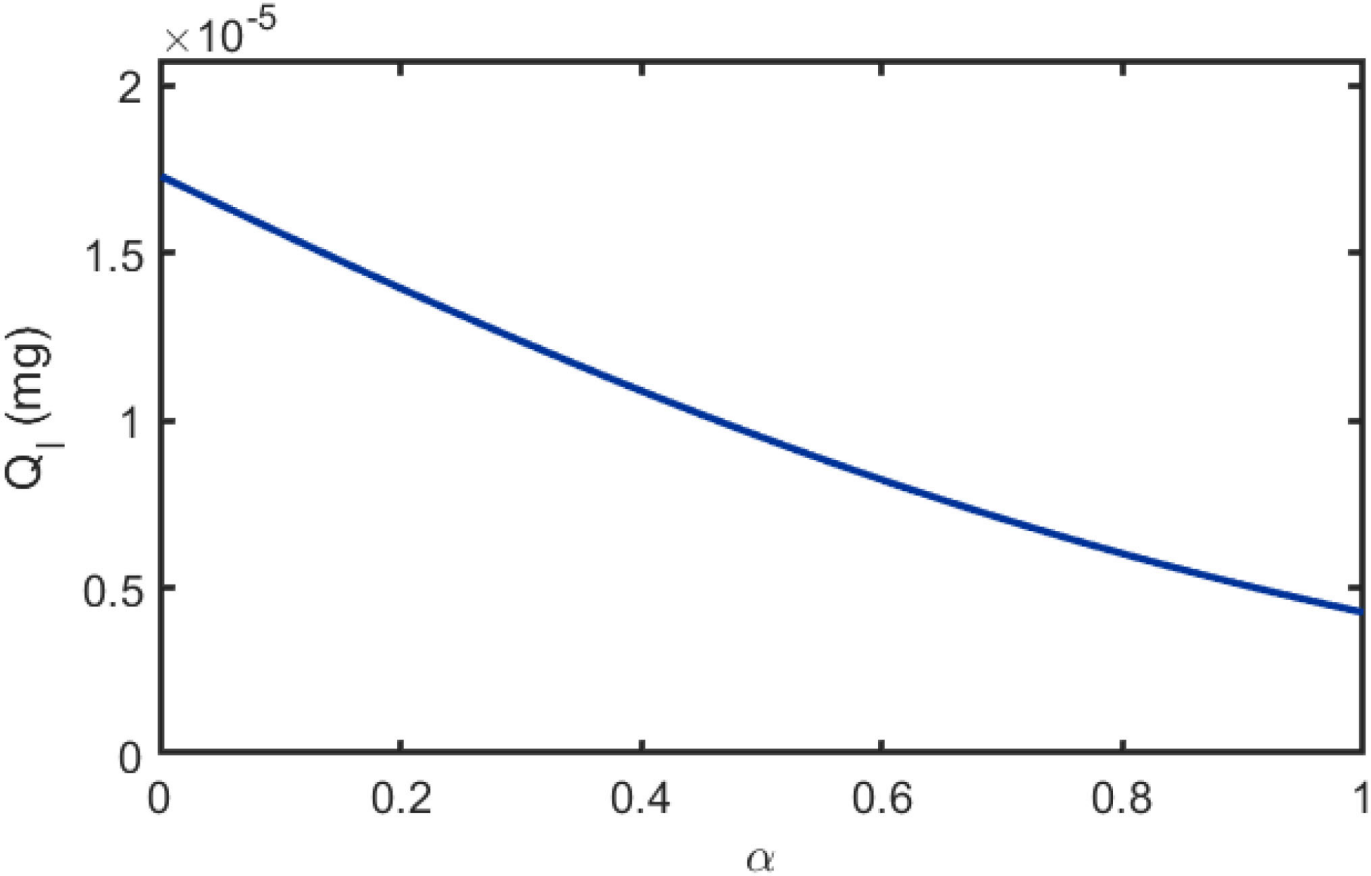
Relationship between the radius ratio *α* and the cumulative received quantity.

Next, we construct a parametric form for *I*(*α,β*). As illustrated in **Figure 3**, the influence of *α* on BVOCs dispersion can be represented by an exponential attenuation model. An increase in *α* indicates a larger interfering plant relative to the receptor plant, implying stronger interception and uptake of BVOCs and thus a reduced cumulative quantity received, *Q_I_*. The rapid-decay property of the exponential function captures the physical process whereby the blocking effect strengthens quickly as the relative size of the interfering plant increases. Accordingly, we define the response function in this scenario as Equation 45:

(45) $$f_{\alpha }=\text{exp }(-\xi \alpha ),$$

where, ξ is an empirical parameter that quantifies the magnitude of attenuation in the flux induced by a unit change in relative size.

**Figure 4:**
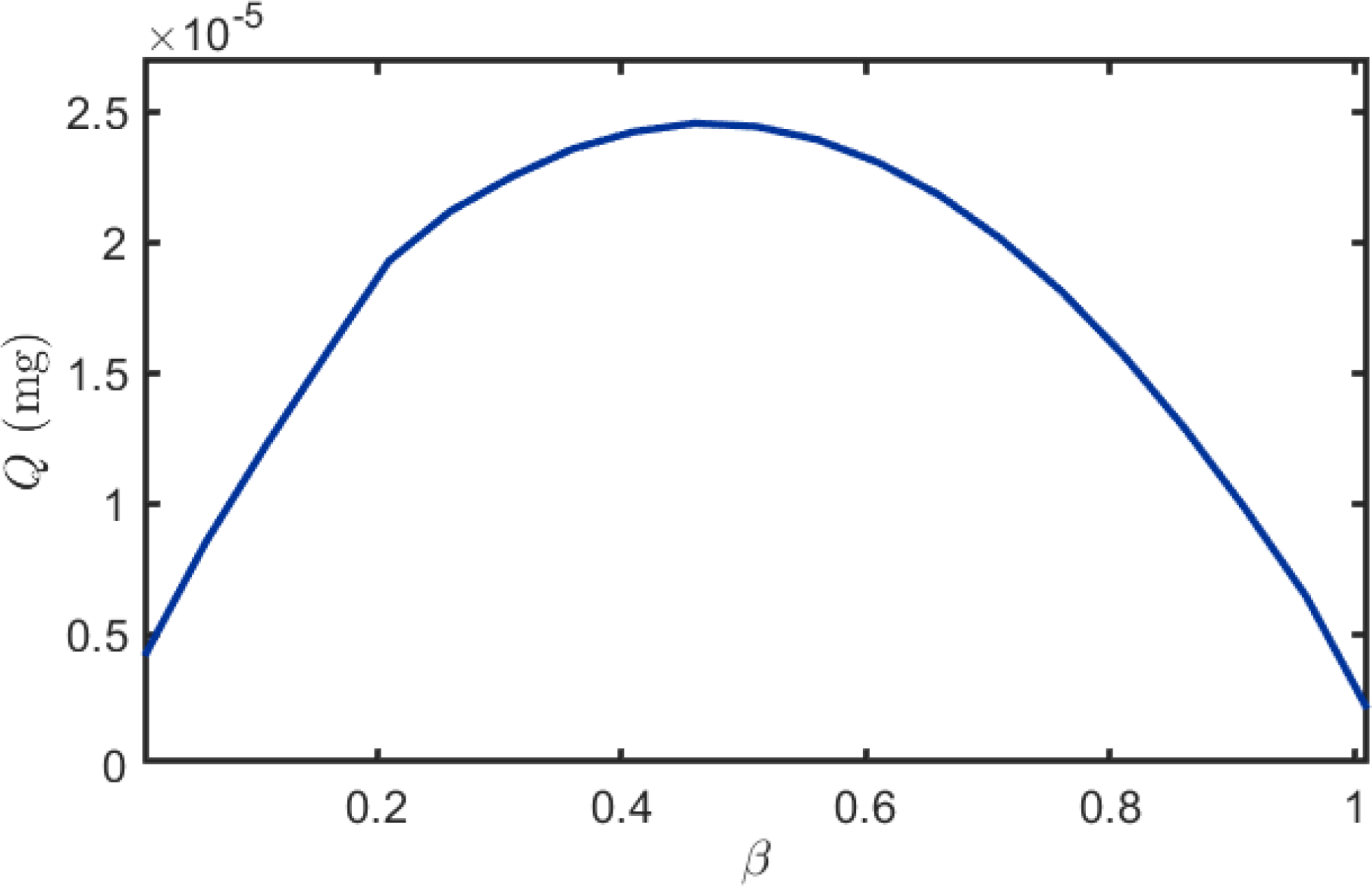
Relationship between the distance ratio *β* and the cumulative received quantity.

The distance between the interfering and receiver plants is a key determinant of BVOCs uptake. As shown in **Figure 4**, *Q_I_* decreases when *β* is small because the interferer lies near the transmitter and over-intercepts BVOCs, and it also decreases when *β* is large because the interferer lies near the receiver and disrupts reception. An intermediate *β* yields the strongest facilitative modulation and maximizes *Q_I_*. Accordingly, we model this positional effect with a Gaussian response function, consistent with Gaussian formulations in atmospheric dispersion and ecological response modeling when an intermediate optimum is observed (Snoun et al., 2023). Note that *β* does not represent atmospheric distance-decay, which is already captured by the baseline no-interference field; rather, it parameterizes the interferer’s relative placement along the path and its additional attenuation of cumulative flux. The corresponding response function is therefore given by Equation 46:

(46) $$f_{\beta }=\lambda \text{exp }(-\frac{{\left(\beta -\mu \right)}^{2}}{\sigma^{2}}),$$

where *λ* is the attenuation constant, *µ* is the peak value, and *σ* is the standard deviation.

Next, under the simplifying assumption that coupling between the size and positional effects of the interfering plant can be neglected, the two effects are treated as independent and additive in their impacts on BVOCs transport. Therefore, Equations 45 and Equations 46 can be combined in a multiplicative form as follows Equation 47:

(47) $$I(\alpha ,\beta )=\lambda \text{exp }(-\xi \alpha )\text{exp }(-\frac{{\left(\beta -\mu \right)}^{2}}{\sigma^{2}})$$

In this equation, *λ*, *µ*, *σ*, and *ξ* are treated as tuning parameters to ensure that the model accurately represents the specific transport scenario. These parameter values are estimated using the Levenberg–Marquardt (LM) curve-fitting method (Tchakpedeou et al., 2022). In addition, to ensure that interference acts only as an attenuating effect, we impose the constraint *λ <* 1 during fitting, thereby guaranteeing 0 *< I*(*α,β*) *<* 1. Consequently, the explicit form of *Q_I_* in Equations 48 can be obtained as Equation 48:

(48) $$\begin{array}{ll} Q_{I} & =\int_{0}^{\tau }({∭}_{V}C_{r}(x,y,z,t,\alpha ,\beta )\text{ d}V)\text{ d}t\\ =\int_{0}^{\tau }({∭}_{V}\lambda C(x,y,z,t)\text{exp }(-\xi \alpha )\text{exp }(-\frac{{\left(\beta -\mu \right)}^{2}}{\sigma^{2}})\text{exp }(-A\text{exp }(-\frac{E_{a}}{RT})t)\text{ d}V)\text{ d}t. \end{array}$$

### 2.4 Channel capacity

In this section, we evaluate the maximum information capacity that plant-to-plant BVOCs transmission can support from a channel-capacity perspective. Building on the preceding analyses of transmission distance, wind-field characteristics, temperature conditions, and canopy-height matching, we conceptualize inter-plant chemical communication as a distributed channel jointly shaped by multiple noise sources and loss mechanisms. Specifically, the BVOCs emission pattern corresponds to the transmitter’s encoding strategy; advection–diffusion, chemical degradation, and gravitational deposition constitute the major sources of interference and attenuation; and the receiver plant’s BVOCs uptake and perception process is analogous to the decoding stage. It should be emphasized that this information-theoretic abstraction is adopted as an engineering-inspired analytical tool for systematic comparison, rather than as a claim that plants have evolved to optimize channel capacity in the strict Shannon sense. Moreover, we note that the conditional Poisson model employed below implicitly assumes that molecular arrivals are mutually independent given the input and that the channel statistics remain approximately stationary within each observation window. In practice, residual molecules from preceding emissions may introduce temporal dependence, and dynamic environmental fluctuations could violate strict stationarity. Therefore, the capacity values derived here should be interpreted as idealized benchmarks under quasi-stationary, memoryless channel assumptions, rather than exact capacity limits of a fully realistic atmospheric channel.

By introducing the concept of channel capacity, we establish a quantitative upper bound on the information transmission rate under different environmental conditions and structural configurations. In ecological terms, the capacity metric quantifies the maximum number of distinguishable signaling states, such as distinct BVOCs concentration levels corresponding to different stress types or intensities that can be reliably discriminated by a receiver plant per unit time under given environmental conditions. This framework enables a systematic comparison of how different environmental parameters constrain or facilitate the fidelity of inter-plant chemical communication, without implying that plants actively maximize information theoretic throughput. Based on conditional probabilities, the mutual information *I*(*X*;*Y)* can be defined as Equation 49:

(49) I(X;Y)=H(Y)−H(Y|X),

where, H(Y) denotes the output entropy, representing the quantity of BVOCs received by the receiver plant over a given time interval, and H(Y|X) denotes the conditional entropy. The latter captures the residual uncertainty in the output *Y* given the input *X*, arising from various interfering factors.

To quantitatively characterize and incorporate the overall uncertainty introduced by different sources of interference within a tractable statistical framework, we adopt a conditional Poisson model to describe the stochasticity at the receiver. Specifically, for a given input intensity, the output fluctuates around its expected value following Poisson-type randomness. Accordingly, when the transmitted BVOCs quantity is *m*, the mean quantity received at the receiver can be expressed as Equation 50:

(50) ξ(m)=mη(x,t,T),

where, η(x,t,T) denotes the transmission efficiency, which aggregates the effects of various interference factors into the average probability that an emitted BVOCs molecule is “successfully received and counted” by the receiver plant. According to Equations 48, we can obtain the total quantity of BVOCs received by the receiver plant over a time interval *t*. Therefore, the transmission efficiency can be defined as the mean received quantity per unit quantity emitted Equation 51:

(51) $$\eta (x,y,z,t,\alpha ,\beta )=\frac{Q_{I}}{m}=\frac{1}{m}\int_{0}^{\tau }({∭}_{V}C_{r}(x,y,z,t,\alpha ,\beta )\text{ d}V)\text{d}t,$$

Building on this, we can further derive the conditional distribution of the absorption quantity at the receiver plant. Specifically, when the input *m* is fixed, the randomness in the output *Q* is entirely induced by various interference factors within the channel. Hence, conditioned on *m*, *Q* follows a Poisson distribution with intensity parameter *ξ*(*m*), and its conditional probability mass function is given by Equation 52:

(52) $$P(Q|m)=\frac{{\left[\xi (m)\right]}^{Q}}{Q!}\text{exp }(-\xi (m)),$$

With the conditional distribution derived above, we can further characterize the receiver’s overall uncertainty, namely the output entropy *H*(*Y)*. During the communication process, the transmitter selects whether to release BVOCs and at what intensity according to a prescribed prior distribution. Consequently, the observed *Q* at the receiver is, in effect, a weighted mixture over different input intensity levels *m*. Therefore, the output entropy can be expressed as Equation 53:

(53) $$H(Y)=-\sum_{Q=0}^{\infty }P(Q)\log_{2}P(Q),$$

where, the marginal distribution *P*(*Q*) is obtained by averaging the conditional distribution P(Q|m) over all possible inputs *m* according to the input prior *P*(*m*), yielding Equation 54:

(54) $$P(Q)=\sum_{m}P(Q|m)P(m),$$

where *P*(*m*) denotes the probability that the input random variable *X* takes the value *m*, namely, the prior distribution of the quantity of BVOCs emitted by the transmitter plant. Next, the conditional entropy *H*(*Y* |*X*) can be written as Equation 55:

(55) $$H(Y|X)=\sum_{m}P(m)\;H(Y|X=m),$$

In the Poisson channel, the output distribution conditioned on a given *m* is Poisson. When the mean received count *ξ*(*m*) lies in the moderate-to-high counting regime, the Poisson distribution can be well approximated by a Gaussian distribution whose variance equals its mean. Accordingly, the conditional entropy can be approximated in closed form via this Gaussian approximation, yielding Equation 56.

(56) $$H(Y|X=m)\approx \frac{1}{2}\log_{2}(2\pi e\xi (m)),$$

Finally, by the definition of channel capacity, it can be expressed as the maximum mutual information over all admissible input distributions Equation 57:

(57) Cap=max I(X;Y).

This capacity provides an idealized upper-bound estimate of the rate at which distinct chemical signals can be reliably distinguished by receiver plants under the quasi-stationary and memoryless assumptions adopted in our Poisson channel model. In practical ecological terms, rather than suggesting that plants actively maximize bit rate, this metric serves as a comparative benchmark to assess how variations in environmental conditions differentially constrain the reliability and distinguishability of inter-plant BVOCs signaling.

## 3 Result

In this section, to examine how model parameters influence BVOCs transport, we performed numerical simulations in MATLAB R2022b. Specifically, we investigated the effects of key factors—such as separation distance, wind speed, and temperature—on the quantity absorbed by the receiver plant, as well as the spatial distribution of BVOCs concentration in air after emission. We further evaluated the fitting accuracy of the interference-plant model and assessed how variations in *α* and *β* affect overall model performance. Finally, we constructed an experimental testbed to provide preliminary validation of the proposed integrated model. To ensure the reliability of the model, **Table 1** summarizes the key system parameters used in the analysis, including atmospheric pressure *P*, the molar mass of air *M*, the universal gas constant *R*, and absolute temperature *T* (Wang and Wang, 2021; Crous et al., 2022); BVOCs density *ρ_b_* (Bai, 2021); the pre-exponential factor *A* and activation energy *E_a_* (Nagawkar et al., 2025).

**Table 1:** Parameters settings.

Parameter	Value
Atmospheric pressure (*P*)	101325Pa
Molar mass of air (*M*)	0.029kg*/*mol
Gas constant for air (*R*)	8.314J*/*(mol · K)
Absolute temperature (*T*)	293.15K
Gravitational acceleration (*g*)	9.81m*/*s^2^
Density of BVOCs (*ρ_b_*)	1.26kg*/*m^3^
Pre-exponential factor of the reaction (*A*)	1 × 10^10^
Activation energy of the reaction (*E_a_*)	50000J*/*mol

### 3.1 Environmental factors analysis

#### 3.1.1 Concentration field analysis

The BVOCs concentration field in air characterizes the spatial evolution of the signal as it propagates from the source to the receiver. Quantitative analysis of its spatiotemporal features enables a direct characterization of BVOCs enrichment and attenuation across locations, thereby providing a basis for subsequent evaluation of the effective absorption flux at the receiver, signal reachability, and sensitivity to environmental factors.

**Figure 5:**
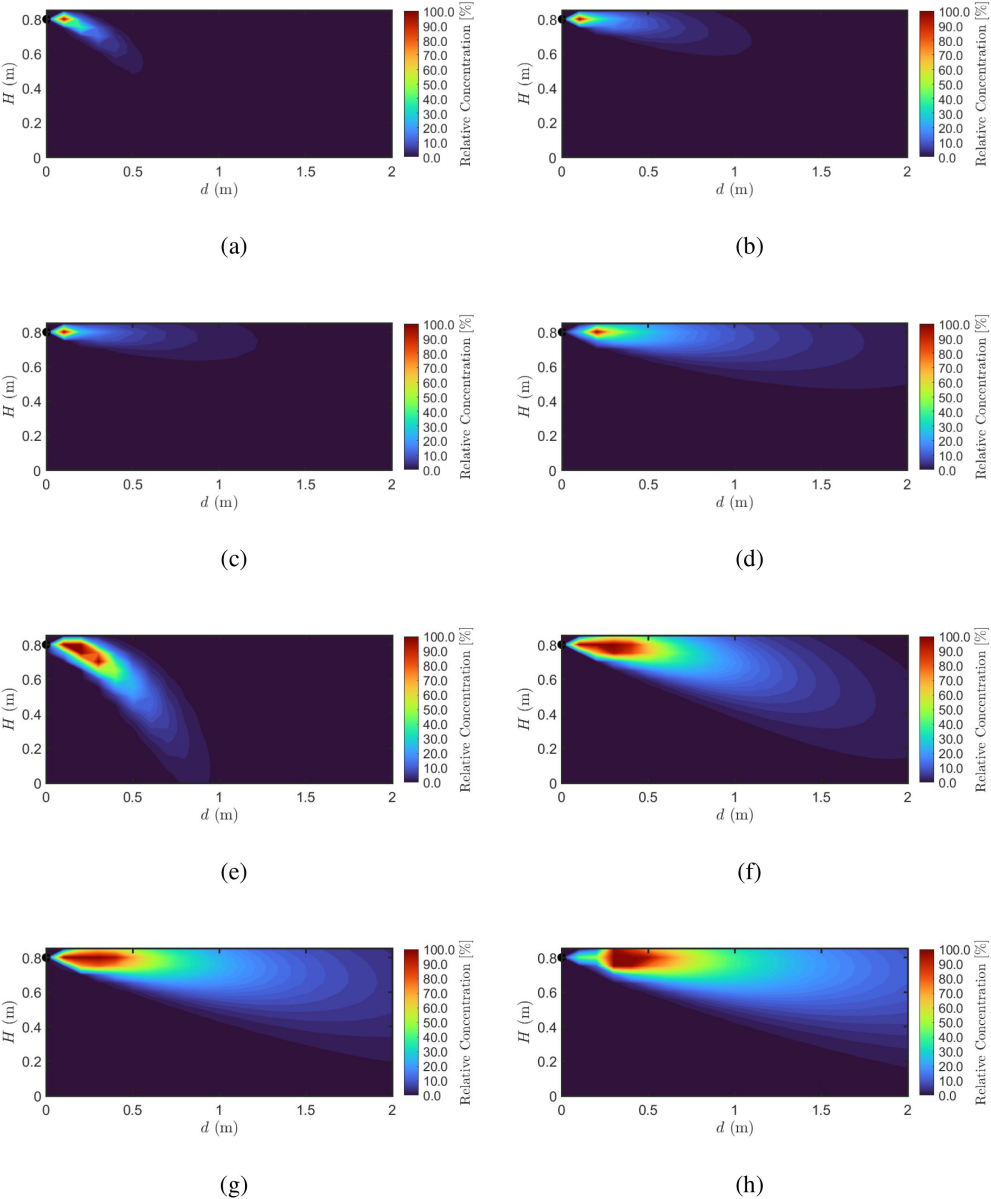
Concentration field distribution of BVOCs in an air channel under varying wind speeds: **(a, e)** *u* = 1 m/s, **(b, f)** *u* = 3 m/s, **(c, g)** *u* = 5 m/s, and **(d, h)** *u* = 7 m/s.

**Figure 5** illustrates the spatial distribution of the BVOCs concentration field emitted from the transmitter plant under different wind speeds for both the OOK-modulated pulsed release (**Figures 5a–d**) and the continuous release (**Figures 5e, h**). While the macro-scale morphology of these concentration fields shares visual similarities with classical atmospheric dispersion models (e.g., Gaussian plume), our molecular communication framework shifts the focus from steady-state mass transport to evaluating the viable “information-carrying footprint” for neighboring plants.

For the OOK-modulated release, at a wind speed of 1 m/s (**Figure 5a**), horizontal advection is weak, and the BVOCs plume is primarily influenced by gravitational settling and buoyancy effects, resulting in a localized, high-concentration signaling zone near the source with limited spatial reach. As wind speed increases to 3 m/s (**Figure 5b**) and 5 m/s (**Figure 5c**), advective transport reshapes the communication channel, leading to a significant elongation of the plume. This directional shift moves the high-concentration core further downstream, indicating a marked enhancement of the effective communication range under moderate wind conditions. When the wind speed reaches 7 m/s (**Figure 5d**), the signal footprint achieves its maximum horizontal coverage; however, increased turbulent mixing and dilution significantly reduce overall relative concentration levels, potentially causing the signal strength to drop below the receiver’s decoding threshold.

To further quantify the distinction between pulsed and continuous emission regimes, **Figures 5e, h** present the concentration fields under continuous release at the same four wind speeds. In contrast to the OOK case, where each discrete pulse undergoes three-dimensional diffusion and decays rapidly in both space and time, the continuous release sustains a steady molecular injection that produces a quasi-steady-state plume envelope with substantially broader spatial coverage. Specifically, at *u* = 1 m/s (**Figure 5e**), the continuous-release plume exhibits a pronounced downward curvature due to the heavy-gas nature of BVOCs, with detectable concentrations extending well beyond the range observed in the corresponding OOK case (**Figure 5a**). As wind speed increases to 3 m/s (**Figure 5f**) and 5 m/s (**Figure 5g**), the gravitational settling is progressively suppressed by horizontal advection, and the plume trajectory flattens while maintaining a significantly longer high-concentration core than its OOK counterpart. At *u* = 7 m/s (**Figure 5h**), horizontal advection dominates, producing a nearly horizontal plume that retains measurable concentration levels across the entire 2 m domain—a stark contrast to the rapidly attenuated pulsed signal in **Figure 5d**.

#### 3.1.2 Distance analysis

**Figure 6:**
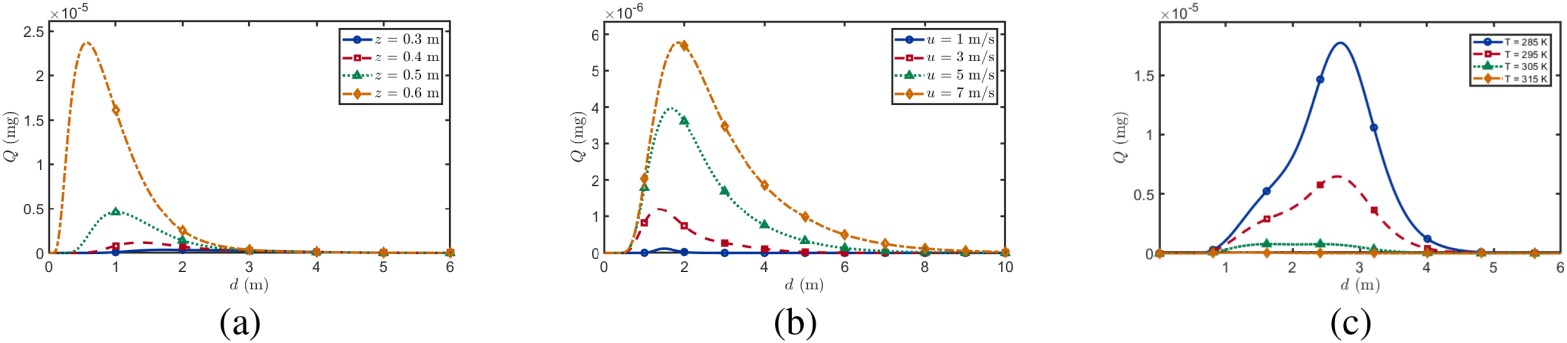
BVOCs uptake as a function of horizontal distance under different environmental conditions. **(a)** Effect of receptor height (*z* = 0.3 − 0.6 m) at constant wind speed (*u* = 3 m/s) and temperature (*T* = 295 K). **(b)** Effect of wind speed (*u* = 1 − 7 m/s) at constant receptor height (*z* = 0.6 m) and temperature (*T* = 295 K). **(c)** Effect of temperature (*T* = 285 − 315 K) at constant receptor height (*z* = 0.6 m) and wind speed (*u* = 3 m/s).

In this section, we analyze the impact of transmitter–receiver distance d on BVOCs absorption. As shown in **Figure 6**, with increasing distance, BVOCs absorption at the receiver initially increases and then decreases, exhibiting a unimodal trend. While this behavior fundamentally reflects the expected physical balance between advective transport and chemical decay, within the molecular communication framework, it quantitatively delineates the spatial boundaries for effective inter-plant signaling. At short distances, downwind advection enhances BVOCs enrichment, facilitating the accumulation of a decipherable signal flux. However, as distance further increases, turbulent dilution and gas-phase oxidation progressively attenuate the chemical signal, causing absorption to decline below viable detection thresholds, ultimately resulting in communication outage.

**Figure 6a** shows BVOCs uptake as a function of horizontal distance at different receptor heights. Using tomato as a representative species, which has a typical canopy height of approximately 0.8 m (Zhu et al., 2025), receptor heights of 0.3–0.6 m are selected to span the lower to mid-upper canopy. Ecologically, this spatial filtering mechanism suggests that receiver positioning dictates signal acquisition. At *z* = 0.6 m, the peak uptake is higher and occurs closer to the source, serving as a highly efficient, short-range signaling link with minimal oxidative depletion. Conversely, at *z* = 0.3 m, the peak is weaker and shifted farther downwind due to prolonged transport and greater chemical loss, indicating a delayed but spatially extended reception strategy.

**Figure 6b** presents BVOCs uptake as a function of horizontal distance under different wind speeds. With increasing distance, the uptake exhibits the characteristic unimodal pattern. Rather than merely showing dispersion, this demonstrates how environmental forcing stretches or compresses the optimal communication range. Stronger winds (*u* = 7 m/s) rapidly transport the plume downwind, rescuing the chemical signal from oxidative decay during transit. Consequently, horizontal advection shifts the highest peak uptake to greater downwind distances, effectively extending the plant’s communicative reach before enhanced dispersion ultimately reduces the signal below viable thresholds.

**Figure 6c** shows BVOCs uptake as a function of distance at different temperatures (285–315 K), covering conditions from temperate growing seasons to extreme summer heat (Esper et al., 2024). Here, the unimodal curve illustrates the vulnerability of the communication channel to thermal-induced signal degradation. At 285 K, the “sweet spot” for signal reception is robust, peaking at approximately 2.5 m from the source. At 295 K, the peak drops markedly and shifts closer to the source, shrinking the effective communication range. Critically, at 305 K and 315 K, uptake remains near zero across all distances. This provides a quantitative, mechanistic explanation for an important ecological phenomenon: under severe heat stress, accelerated gas-phase oxidation destroys the BVOCs before they can reach neighboring plants, effectively “silencing” chemical communication within the canopy.

#### 3.1.3 Temperature analysis

Temperature is a key environmental factor governing the atmospheric transport efficiency of BVOCs. In this subsection, we systematically examine how ambient temperature modulates BVOCs uptake at the receptor under different wind speeds.

**Figure 7:**
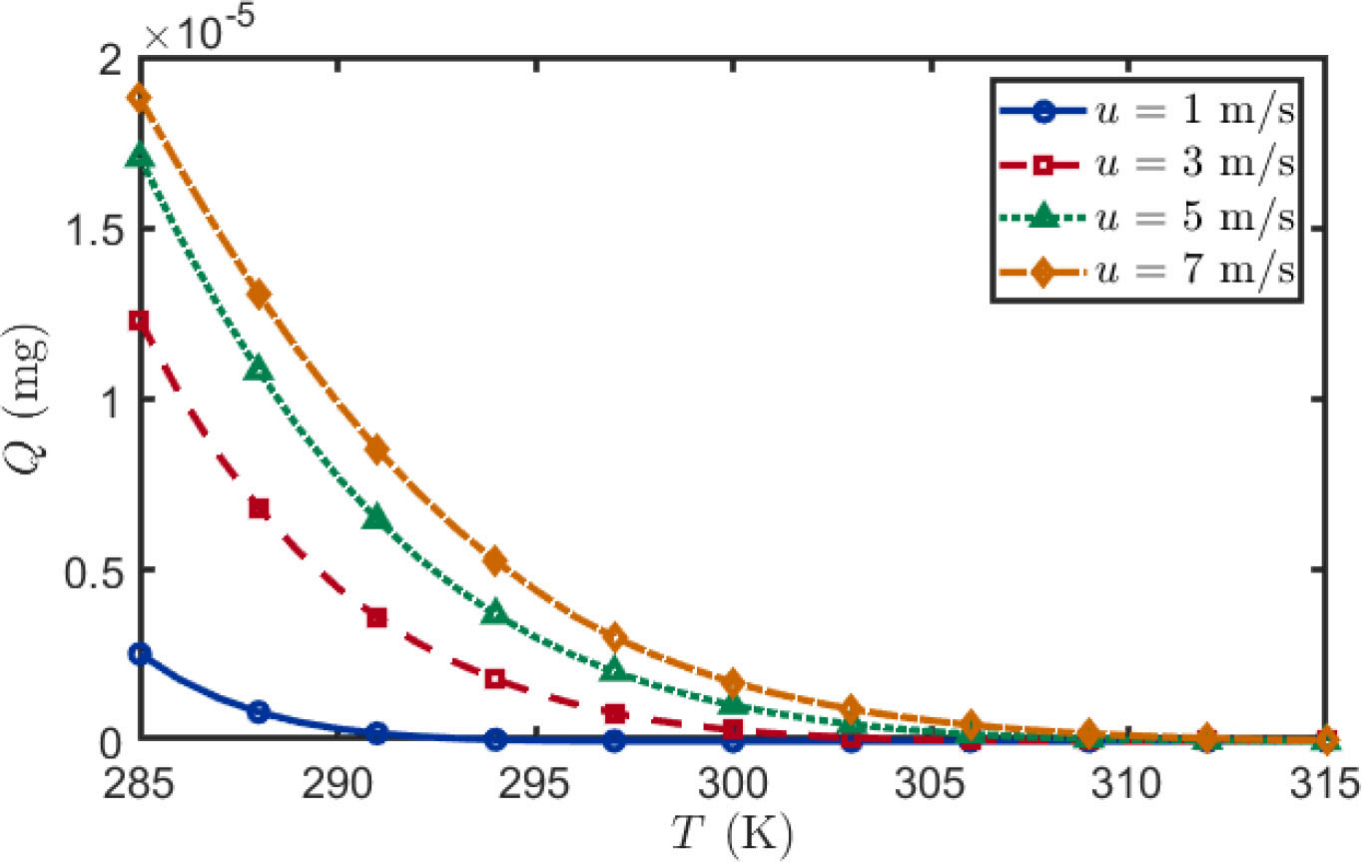
Effect of temperature on BVOCs uptake as a function of wind speed. BVOCs uptake decreases monotonically with increasing temperature for all wind speeds. At low temperatures (285 K), high wind speed (7 m/s) substantially enhances uptake compared to low wind speed (1 m/s). However, as temperature increases above 305 K, all curves converge near zero, indicating that chemical oxidation overwhelms advective transport at elevated temperatures. Emitter height *H* = 0.8 m, receptor height *z* = 0.6 m, horizontal distance *d* = 1 m.

As shown in **Figure 7**, BVOCs uptake decreases monotonically with increasing temperature under all wind-speed conditions, but the decline rate is strongly wind-speed dependent. At high wind speed (7 m/s), uptake is markedly higher than at low wind speed (1 m/s) because faster horizontal advection shortens the transit time, effectively reducing the cumulative effect of chemical oxidation and turbulent dilution during transport. As temperature increases to 295 K, uptake drops sharply for all wind speeds, and the relative advantage of high wind speed progressively diminishes. At temperatures above 305 K, uptake becomes negligible across all wind speeds, and all curves converge near zero. This convergence reflects the dominant role of accelerated photochemical oxidation at elevated temperatures: the oxidation rate increases so rapidly that chemical loss overwhelms advective transport, rendering wind speed variations inconsequential. Under these conditions, nearly all emitted BVOCs are consumed within the near-source region before reaching the receptor.

#### 3.1.4 Analysis of plant height at transmitting and receiving plants

**Figure 8:**
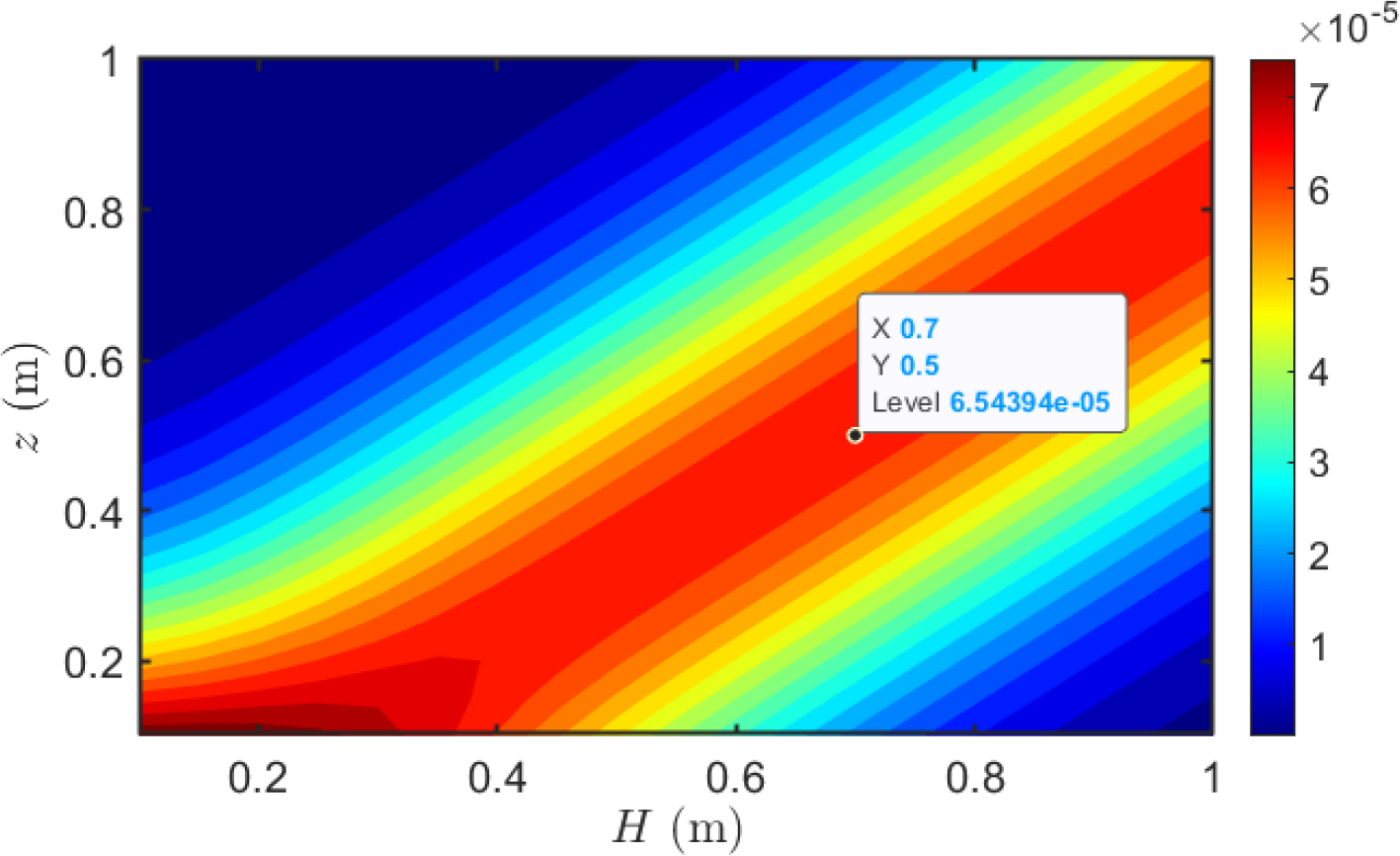
BVOCs uptake as a function of emitter and receiver heights at fixed horizontal distance. The contour plot shows BVOCs uptake in the emitter height (*H*)–receiver height (*z*) plane at a horizontal separation of 1 m. Maximum uptake (red region) occurs along a diagonal band where the receiver is positioned at approximately 70% of the emitter height (*z/H* ≈ 0.7). This optimal height ratio remains constant as emitter height increases, indicating a robust vertical matching criterion for effective inter-plant BVOCs communication. Wind speed: *u* = 3 m/s; temperature: *T* = 295 K.

This section examines how the relative heights of transmitter and receiver plants influence BVOCs uptake. As shown in **Figure 8**, at a horizontal separation of 1 m, BVOCs uptake is highest along a diagonal band where the receiver height is slightly below the emitter height. Uptake peaks at a receiver-to-emitter height ratio of *z/H* ≈ 0.7 under the current baseline simulation parameters. In this configuration, the slightly lower receiver intercepts the high-concentration plume core after it has descended from the emission point but before significant lateral dispersion has reduced the concentration. Conversely, when the receiver height substantially exceeds the emitter height, BVOCs uptake becomes negligible. Under these conditions, the emitted plume is predominantly transported downward and laterally by gravitational settling and horizontal advection, while upward vertical mixing is insufficient to deliver significant BVOCs concentrations to the elevated receptor.

As emitter height increases, the high-uptake band shifts to higher absolute receptor heights. While the optimal relative configuration remains relatively consistent within our specific modeling framework, we explicitly acknowledge that this *z/H* ≈ 0.7 ratio is model-dependent. This theoretically derived height ratio indicates a vertical height-matching window for plant-to-plant communication, governed by plume structure, turbulent mixing, and gravitational settling. Within this height-matching window, the receiver is optimally positioned to intercept the descending plume where the BVOCs concentration remains high, while transport time is short enough to minimize oxidative and dispersive losses. However, it must be emphasized that this optimal ratio should be interpreted as a baseline heuristic rather than an absolute ecological constant. In real-world canopies, environmental uncertainties will inevitably shift plume trajectories. Consequently, without a comprehensive probabilistic uncertainty analysis, the actual optimal height-matching configuration in the field is expected to exhibit considerable variability driven by local micrometeorological fluctuations.

#### 3.1.5 Propagation time analysis

**Figure 9:**
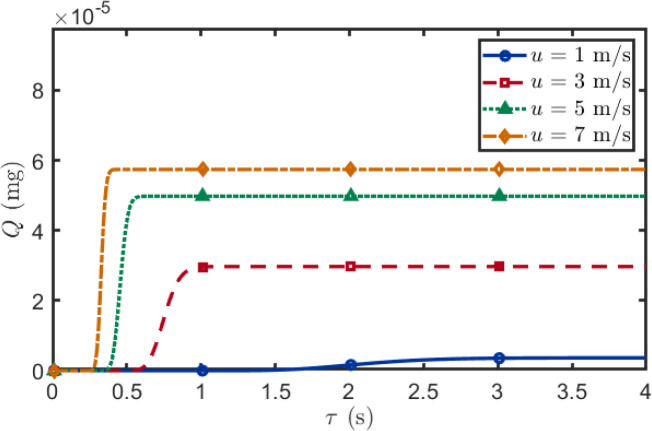
Temporal evolution of cumulative BVOCs uptake under different wind speeds. Higher wind speeds result in both faster signal arrival and greater total BVOCs accumulation. At high wind speed (7 m/s), uptake reaches plateau within 0.5 s, whereas at low wind speed (1 m/s), accumulation is substantially slower and reaches a lower plateau. Emitter height: *H* = 0.8 m; receptor height: *z* = 0.6 m; horizontal distance: *d* = 1 m; temperature: *T* = 295 K.

This section examines the temporal dynamics of BVOCs uptake and the effect of wind speed on signal reception time. As shown in **Figure 9**, when the horizontal transmitter–receiver distance is fixed at 1 m, the cumulative BVOCs uptake at the receiver exhibits distinct temporal patterns under different wind speeds. At high wind speed (7 m/s), the receiver rapidly accumulates BVOCs within approximately 0.5 s. In contrast, at low wind speed (1 m/s), the accumulation process is much slower, requiring over 3 s to approach a substantially lower plateau of 0.5. These differences arise from two interacting factors. First, higher wind speeds reduce the transit time, enabling earlier signal arrival. Second, shorter transit times minimize cumulative oxidative loss during transport, resulting in higher total BVOCs delivery to the receiver. From an ecological perspective, the combination of faster signal arrival and higher signal intensity at elevated wind speeds may be critical for effective inter-plant communication.

### 3.2 Analysis of fitting results

This section analyzes the model under conditions with interfering plants, focusing on the fitting accuracy for cumulative uptake *Q* and the effects of radius ratio and distance ratio on model performance. It comprises two parts. The first part describes the simulation setup, detailing parameter configurations and experimental design. The second part presents the fitting results and analysis.

#### 3.2.1 Simulation settings

The simulation parameters are shown in **Table 2**, with fixed parameters given in **Table 1**. The model includes one transmitter and one receiver, with the receiver radius set to 0.15 m to represent small- to medium-sized plants in natural environments. The radius ratio *α* and distance ratio *β* both range within [0,1]. When *α >* 1, the interfering plant is larger than the receiver; when *β >* 1, the interfering plant is positioned beyond the midpoint between transmitter and receiver. These two scenarios have limited relevance to the research context and are therefore excluded from consideration. Additionally, the separation between transmitter and receiver is fixed at 1 m. This distance provides sufficient space for BVOCs dispersion and decay while ensuring the receiver can detect effective molecular signals. Consequently, the numerical simulations realistically capture the chemical communication process between plants and provide a physically sound basis for subsequent analysis of signaling characteristics and information transmission performance under different combinations of *α* and *β*.

**Table 2:** Parameters settings.

Parameter	Value
Release quantities (*m*)	0.0016mg
Radius of absorbing spherical receivers (*R*)	0.15m
Simulation step	0.001s
Simulation duration	2s
Simulation repetitions	100

In terms of temporal discretization, the simulation time step is set to 0.001 s to capture molecular transport dynamics and record molecular positions at each time step for evaluating uptake efficiency at the receiver. The total simulation duration is 2 s, covering the complete process from BVOCs release to uptake by the receiver. To minimize statistical errors arising from stochasticity, simulations are independently repeated 100 times for each parameter configuration, and the average *Q* value is computed accordingly. Furthermore, by systematically varying the radius ratio *α* and distance ratio *β*, a comprehensive comparative analysis of their effects on *Q* is conducted, thereby elucidating the variation patterns of *Q* under different interfering plant scenarios.

#### 3.2.2 Fitting results

In this section, we fit the experimental data to the theoretical equation and compare the numerical results of Equations 48 with the simulation outputs. To rigorously quantify the agreement between the numerical simulations and the analytical solutions, statistical metrics including the coefficient of *R*^2^, SSE and RMSE are evaluated. The numerical results demonstrate a strong quantitative correlation between the simulation outcomes and theoretical predictions.

**Figure 10:**
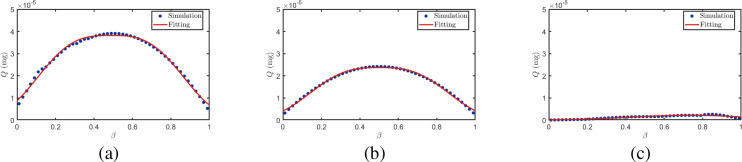
Relationship between BVOCs reception quantity and distance ratio *β*.

**Figure 10** illustrates the relationship between cumulative uptake *Q* and distance ratio *β*. After fitting the data under different *α* values, the resulting curves highlight the overall trend: *Q* initially increases and then decreases with increasing *β*. This phenomenon can be attributed to the interfering plant’s position dependent blocking effect. When positioned near the transmitter, it obstructs BVOCs immediately after release before sufficient dispersion, significantly reducing molecular flux to the receiver. Conversely, when near the receiver, it intercepts airborne BVOCs before arrival, similarly diminishing uptake. In contrast, when positioned midway, the blocking effect is relatively weak, yielding higher *Q* values in the intermediate *β* range. Furthermore, as shown in **Figures 10a–c**, *Q* decreases with increasing radius ratio *α*, as larger interfering plant volumes exert stronger blocking effects on BVOCs, further reducing molecular transmission to the receiver. **Table 3** presents the fitting parameters and corresponding goodness-of-fit metrics for the scenarios depicted in **Figure 10**. Specifically, the fitted results achieve *R*^2^ values of 0.9929, 0.9951, and 0.9912 for *α* = 0.1, 0.5, and 1.0, respectively, with RMSE values of 0.00844, 0.00454, and 0.00243. These results confirm the excellent quantitative agreement between the analytical solutions and the numerical simulations.

**Table 3:** Fitting parameters and performance for **Figure 10**.

*α*	*λ*	*ξ*	*µ*	*σ*	*R* ^2^	SSE	RMSE
0.1	0.5011	0.9804	0.4868	0.2706	0.9929	0.000356	0.00844
0.5	0.4971	1.0521	0.5103	0.2758	0.9951	0.000103	0.00454
1.0	0.5008	0.9842	0.7283	0.4326	0.9912	0.000295	0.00243

**Figure 11:**
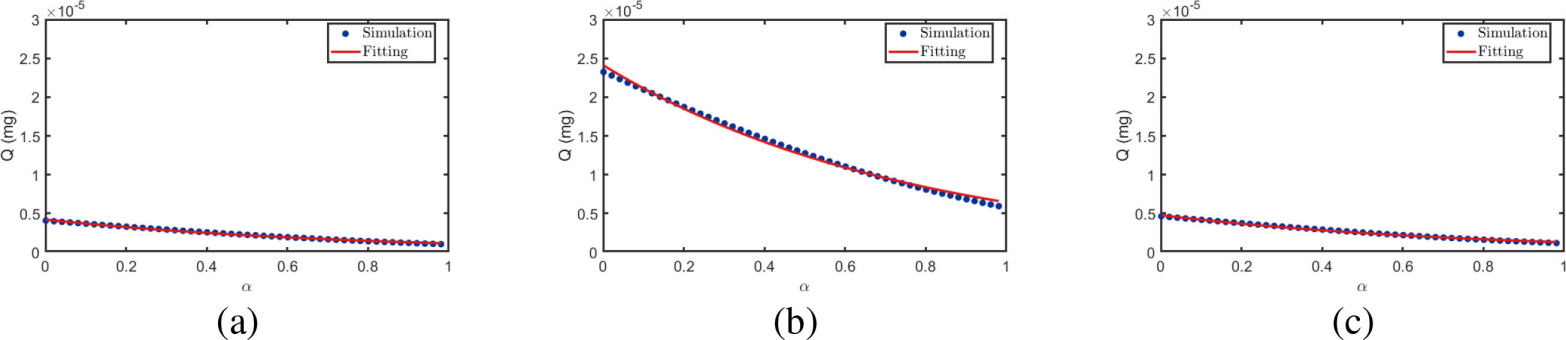
Relationship between BVOCs uptake and radius ratio *α*.

**Figure 11** illustrates the relationship between cumulative uptake *Q* and radius ratio *α*. It is evident that *Q* gradually decreases with increasing *α*. This is because as the interfering plant volume increases, its effective cross-sectional area for intercepting airborne BVOCs also increases, leading to a corresponding increase in the intercepted BVOCs quantity and a sustained reduction in the quantity of BVOCs reaching the receiver plant. Furthermore, as shown in **Figures 11a–c**, as the interfering plant gradually shifts from a position near the transmitter to one near the receiver, *Q* is generally higher when the plant is positioned in the intermediate region compared to when it is near either endpoint. This indicates that the interference strength on BVOCs transmission is relatively weakest when the interfering plant is located at the midpoint position. **Table 4** presents the fitting parameters and corresponding goodness-of-fit metrics for the scenarios depicted in **Figure 11**. Specifically, the fitted results achieve *R*^2^ values of 0.9936, 0.9949, and 0.9947 for *β* = 0.1, 0.5, and 0.9, respectively, with RMSE values of 0.000656, 0.000368, and 0.000744. These results further verify the strong quantitative consistency and robustness of the proposed analytical model under different spatial configurations.

**Table 4:** Fitting parameters and performance for **Figure 11**.

β	*λ*	*ξ*	*µ*	*σ*	*R* ^2^	SSE	RMSE
0.1	1.2189	1.2995	0.5682	0.6732	0.9936	0.0000215	0.000656
0.5	1.2861	1.3201	0.6433	0.7674	0.9949	0.0000678	0.000368
0.9	1.2262	1.3129	0.6135	0.6866	0.9947	0.0000277	0.000744

### 3.3 Channel capacity analysis

This subsection systematically analyzes how channel capacity in BVOCs mediated inter-plant communication depends on key environmental and system parameters. Before examining these dependencies, it is important to clarify the role of the modulation scheme in determining the existence and behavior of channel capacity. Although a continuous constant-flux source can produce the highest steady-state molecular concentration at the receiver, its time-invariant nature conveys no information, resulting in a channel capacity of strictly 0 bps. In contrast, OOK modulation encodes information through discrete emission and silence states, thereby enabling a non-zero channel capacity. At the same time, the transient accumulation and tailing of molecular pulses introduce inter-symbol interference, which fundamentally limits capacity and leads to saturation effects under high SNR or large emission quantities. With this premise established, we examine the effects of emitter-receptor distance, ambient temperature, BVOCs emission quantity, and SNR on channel capacity. These results provide quantitative foundations for optimizing spatial deployment, environmental adaptation, and signaling strategies in plant molecular communication systems.

**Figure 12:**
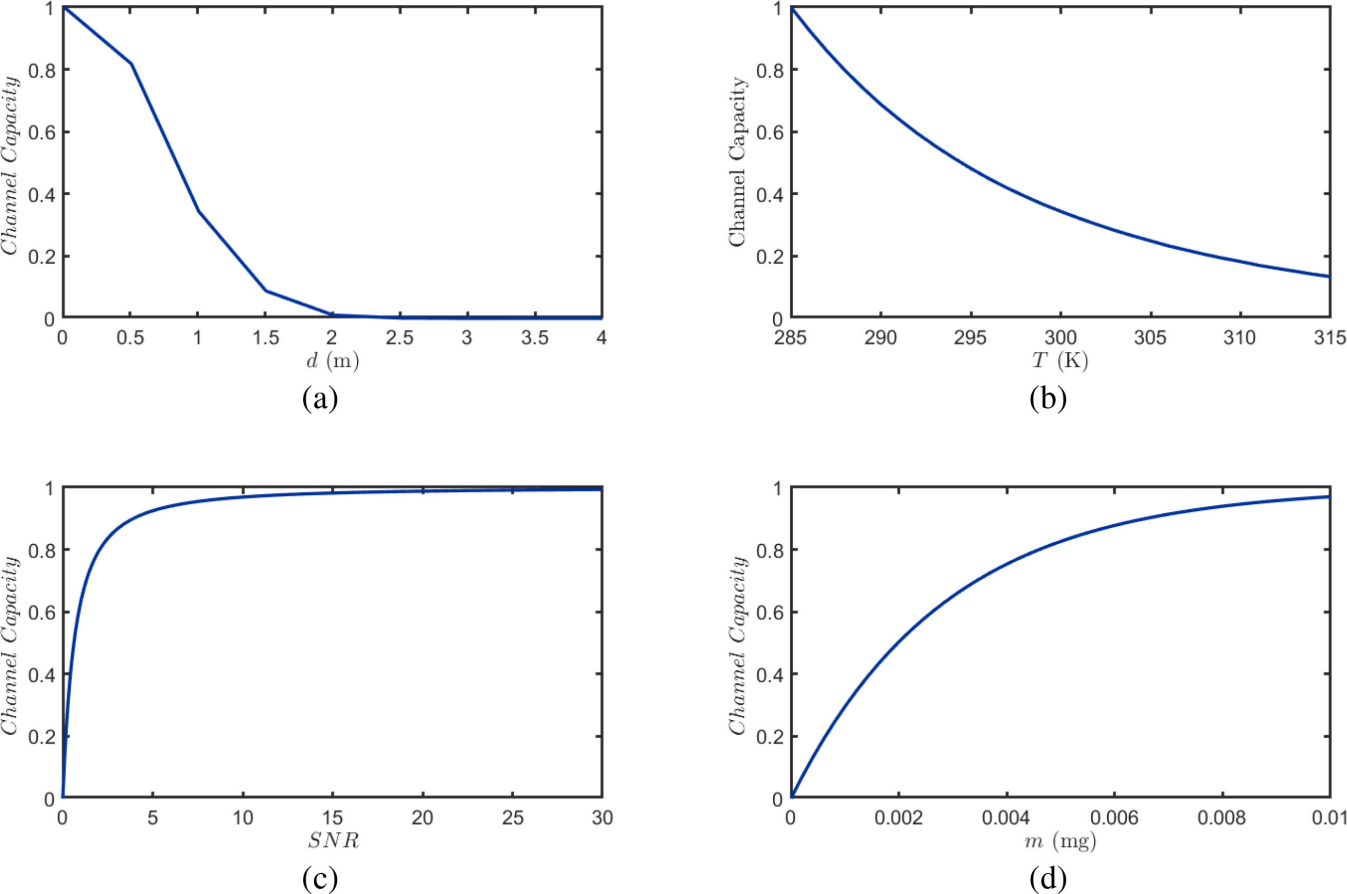
Channel capacity as a function of key system and environmental parameters. **(a)** Effect of emitter–receptor distance *d* at fixed *T* = 295 K and *m* = 0.0016 mg. **(b)** Effect of ambient temperature *T* at fixed *d* = 1 m and *m* = 0.0016 mg. **(c)** Shannon capacity as a function of SNR, showing asymptotic approach to unity for high SNR. **(d)** Effect of BVOC emission rate *m* at fixed *d* = 1 m and *T* = 295 K.

**Figure 12** illustrates the effects of transmitter-receiver distance *d*, ambient temperature *T*, signal-to-noise ratio (SNR), and BVOCs emission quantity *m* on channel capacity. As shown in **Figure 12a**, the channel capacity monotonically decreases with increasing distance, approaching nearly zero when *d* reaches approximately 2 m. This is primarily attributed to the dilution effect caused by turbulent diffusion and the cumulative impact of loss mechanisms such as photochemical oxidation and gravitational settling. **Figure 12b** demonstrates that channel capacity exhibits a monotonic decline with increasing temperature, with capacity attenuating to nearly 0.1 as *T* approaches 315 K. This is mainly because elevated temperatures significantly accelerate the photochemical oxidation reactions of BVOCs and enhance turbulent mixing, resulting in reduced effective concentration differences at the receiver and lower SNR. **Figure 12c** shows that channel capacity monotonically increases with SNR and gradually saturates approaching unity after SNR = 10, reflecting the enhanced signal distinguishability and reduced decision error probability under high SNR conditions. **Figure 12d** indicates that channel capacity monotonically increases with emission quantity *m*, approaching unity as *m* nears 0.01 mg, demonstrating that increasing emission rate can significantly enhance signal concentration and SNR at the receiver. However, due to the influences of photochemical reactions and diffusion dilution, the capacity exhibits an asymptotic saturation trend.

### 3.4 An MC testbed

This section presents the construction of a molecular communication testbed, whose overall design follows the experimental architecture proposed in (Brand et al., 2022). The testbed aims to simulate the propagation process of BVOCs in natural environments and experimentally validate the accuracy of the proposed theoretical model. This platform provides a controlled and reproducible experimental environment, establishing an experimental foundation for further exploration of potential applications of molecular communication in the fields of ecology and environmental science.

#### 3.4.1 Basic parts of the testbed

**Figure 13:**
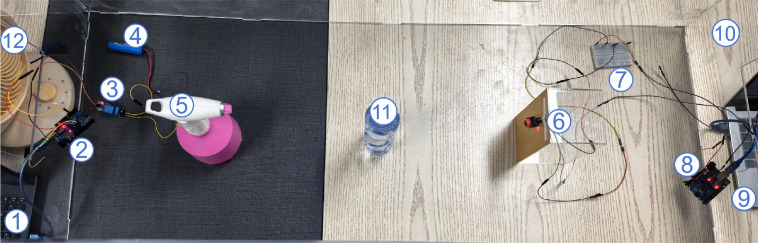
MC testbed. Components: (1) Transmitter Control Computer, (2) Transmitter Arduino Board, (3) Relay Switch, (4) Rechargeable Battery, (5) Electronic Atomizer, (6) MQ-3 Alcohol Sensor, (7) Breadboard, (8) Receiver Arduino Board, (9) Data Recording Laptop, (10) PVC Isolation Box, (11) Bottle, (12) Fan.

**Figure 14:**
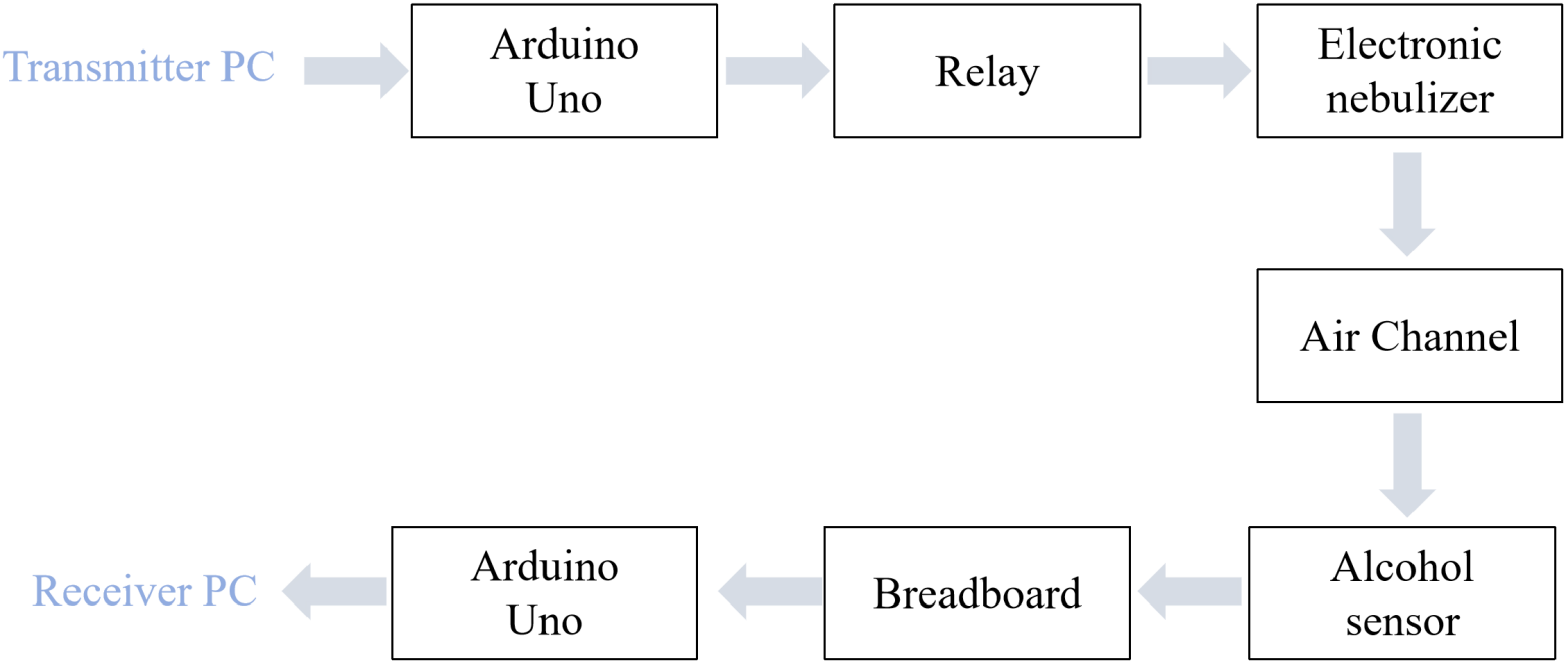
Structure of the MC testing platform.

**Figure 13** presents a photograph of the platform, while **Figure 14** shows the overall structural diagram. Both the transmitter and receiver are placed within a top-open PVC box (10) to minimize the influence of environmental disturbances around the experimental bench on the results. The transmitter consists of a laptop computer (Computer 1), an Arduino development board (Board 2), a relay (Relay 3), a rechargeable solid-state battery (Battery 4), a fan (Fan 12), and an electronic atomizer (Atomizer 5). The electronic atomizer is loaded with an alcohol solution of predetermined concentration to simulate BVOCs emitted by plants under stress conditions. The fan is designed to simulate natural wind conditions and control the airflow environment. The solid-state battery supplies power to the atomizer, while the relay connects the Arduino board (Board 2) to the electronic atomizer via jumper wires, enabling effective control over the atomizer’s on/off state. The other end of the Arduino board is connected to the laptop (Computer 1), on which a control program runs to precisely schedule the emission behavior of the electronic atomizer. Through the computer interface, the atomizer’s operational parameters, such as spray frequency, duration, and emission volume, can be configured, thereby flexibly simulating different environmental and signal transmission scenarios under laboratory conditions.

Between the transmitter and receiver, an empty bottle (Bottle 11) is positioned midway between the atomizer (5) and the alcohol sensor (6) to simulate the presence of an interfering plant that may exist between the emitter and receiver plants in natural plant communities, thereby introducing a physical obstacle effect on signal propagation.

The receiver has a similar configuration to the transmitter, comprising an MQ-3 alcohol sensor (Sensor 6), a breadboard (Breadboard 7), an Arduino development board (Board 8), and a laptop computer (Laptop 9). The breadboard connects the Arduino board to the alcohol sensor via jumper wires. The alcohol sensor identifies alcohol molecules based on the conductivity variation of its internal semiconductor material under different alcohol concentrations, converting this conductivity change into electrical signals to enable real-time monitoring of ambient alcohol concentration. After signal acquisition and preliminary processing, the Arduino board (Board 8) transmits the corresponding alcohol concentration data to the laptop (Laptop 9), where data recording and visualization are performed for subsequent analysis.

#### 3.4.2 Signal transmission and experimentation

In this section, leveraging the constructed testbed, we conduct simulation experiments on the complete communication process of information transmission.

To ensure the validity of the experimental results, an alcohol solution with a volume fraction of 75% is first mixed with water at a volume ratio of 1:3 prior to the experiment, and the resulting mixture is loaded into the electronic atomizer (5) for use. During the experimental configuration phase, the platform employs an OOK-based modulation strategy to simulate the information transmission mechanism between plants in nature. The transmitter PC program utilizes binary encoding to characterize the release state of alcohol molecules: the emission of alcohol molecules is designated as bit “1”, while no emission is designated as bit “0”. When the program input is 1, the atomizer (5) is triggered to release a metered quantity of alcohol vapor; when the input is 0, the atomizer remains in standby mode without performing any spraying operation. The released alcohol propagates through the air channel and eventually reaches the receiver. At the receiver module, the MQ-3 semiconductor alcohol sensor is employed to detect variations in its internal resistance and convert them into voltage signals. Based on the amplitude variations of these electrical signals, the presence or absence of alcohol emission within the current time slot can be determined, thereby enabling the reconstruction of the transmitter’s input signal state.

**Figure 15:**
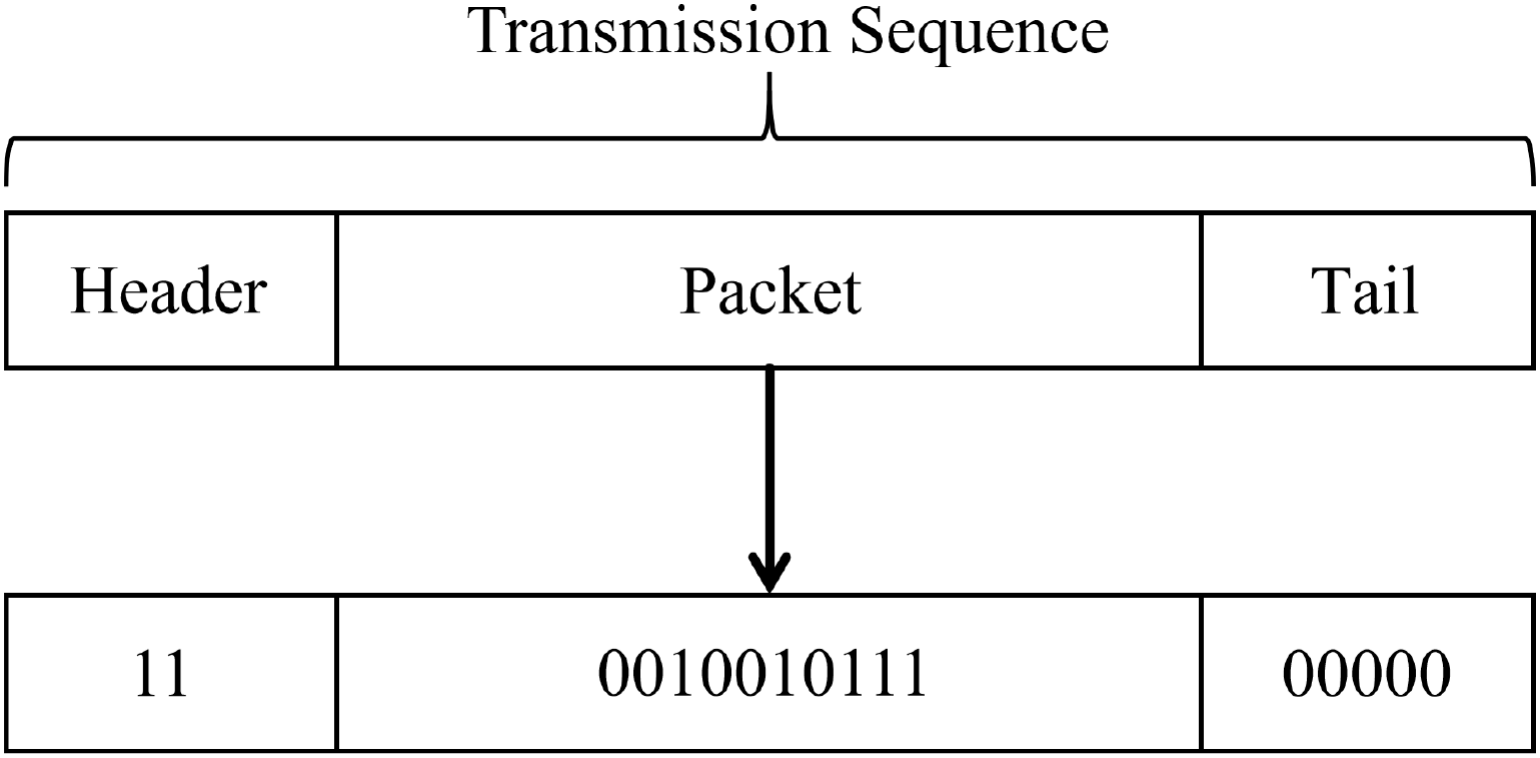
Transmission sequence.

In our testbed, information is encoded using a binary scheme. **Figure 15** illustrates a predefined transmission sequence defined in the transmitter PC. This sequence comprises three components: a frame header, a data packet, and a frame trailer. The frame header, consisting of two consecutive bits “1”, is used to detect whether the information has reached the receiver. The data packet is composed of a 10-bit binary sequence. The frame trailer, consisting of five consecutive bits “0”, ensures that the sensor returns to its initial state, thereby guaranteeing reliable synchronization and correct decoding of signals between the transmitter and receiver. For bit “1”, the atomizer operates for 100 ms followed by a 400 ms interval to simulate a single BVOCs emission event; for bit “0”, the atomizer releases no molecules and maintains a 500 ms idle state.

**Figure 16:**
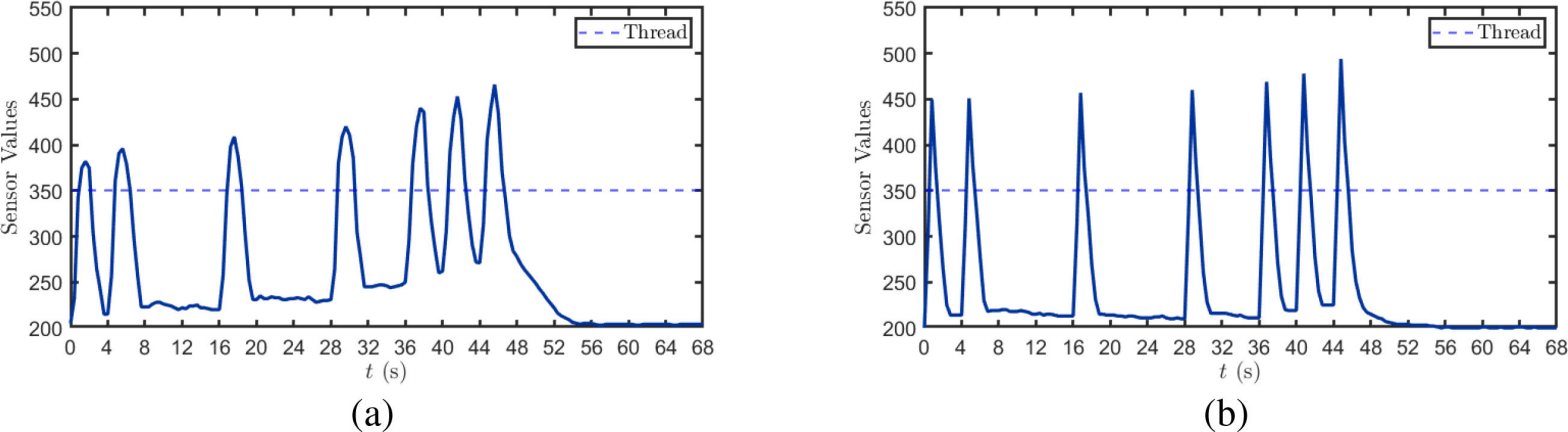
Sensor response characteristics at different wind speeds. **(a)** *u* = 0 m/s, **(b)** *u* = 0.5 m/s.

At the receiver, the arrival of alcohol molecules is determined based on the alcohol sensor readings. As illustrated in **Figure 16**, the experiment employs alcohol sensor readings to identify the arrival of molecular signals. When alcohol molecules are transmitted within a time slot, the tin dioxide sensing material inside the MQ-3 sensor undergoes redox reactions with the alcohol molecules, leading to increased conductivity and decreased resistance, ultimately manifesting as a significant rise in sensor readings. Based on this response mechanism, an empirical threshold of 350 is established, when the real-time reading exceeds this threshold, the receiver is deemed to have successfully detected alcohol molecules from the transmitter.

**Figure 16a** presents the sensor response curve under fan-off conditions, where the detected value rises from 210 to approximately 385 before declining to around 230. During the continuous emission period from 36 to 48 s, the baseline values to which the sensor returns after each decline exhibit a progressively increasing trend, indicating the continuous accumulation of alcohol molecules at the receiver. After cessation of emission at 48 s, the values gradually return to the initial baseline. **Figure 16b** demonstrates the experimental results with a fan introduced to simulate natural wind speed. Under the influence of convective airflow, the sensor reaches peak values within each time slot more rapidly, and exhibits lower recovery values after detection completion, indicating that wind speed accelerates both the transport and dissipation processes of alcohol molecules.

In summary, the alcohol sensor can effectively identify the arrival and departure of molecular signals; however, molecular residue and accumulation effects on the sensor surface compromise the stability of the response baseline. The introduction of convective airflow significantly reduces signal arrival time and promotes molecular dissipation, thereby enhancing the temporal resolution and signal recovery speed of the system.

### 3.5 Theory and model fitting

#### 3.5.1 Distance–concentration relationship under varying wind speeds

This section validates the theoretical model proposed in Chapter 2 based on the constructed molecular communication testbed. The experimental platform employs ethanol (molecular weight 46 g/mol) as the information carrier, whose molecular weight, volatility, and diffusion characteristics are comparable to those of low-molecular-weight BVOCs commonly released by plants (e.g., acetaldehyde, acetone, and methanol). The response time (≤1 s) and sensitivity range (10–1000 ppm) of the MQ-3 sensor at the receiver enable effective capture of concentration dynamics. To better approximate the propagation scenarios of BVOCs in real natural environments, wind speed is selected as the primary research variable. With temperature (*T* = 293 K), relative humidity (RH = 50%), and transmission distance (*d* = 1.5 m) held constant, three wind speed conditions (*u* = 0 m/s, *u* = 0.5 m/s, *u* = 1 m/s) are configured, and the sensor response curves at the receiver are collected.

**Figure 17:**
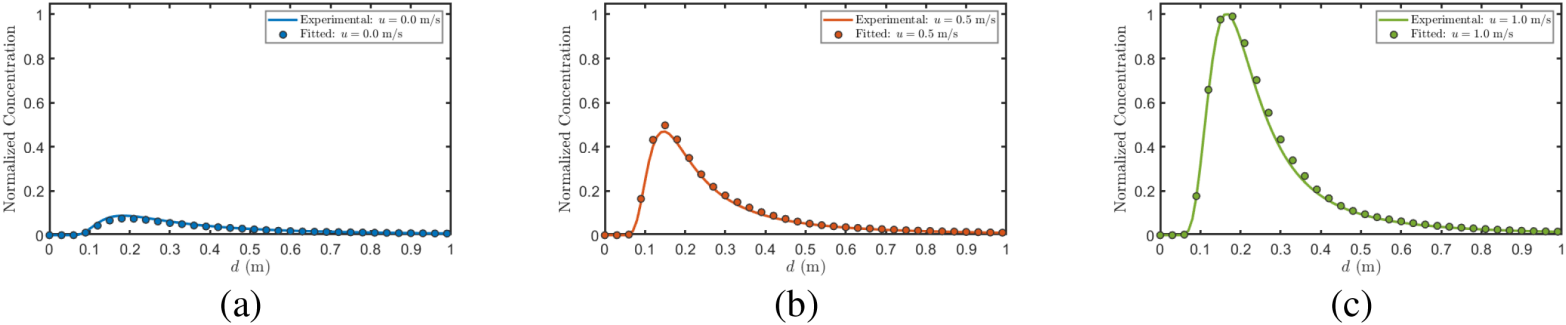
Fitting results at various wind velocities. **(a)** *u* = 0 m/s, **(b)** *u* = 0.5 m/s, **(c)** *u* = 1 m/s.

Given that the theoretical model adopts several idealized assumptions (uniform flow field, neglect of boundary effects), discrepancies inevitably exist between model predictions and actual experimental conditions (including turbulent perturbations, sensor response delay, wall adsorption effects, etc.). To improve fitting accuracy, this section introduces two types of corrections to the original theoretical formula (Equations 43): (1) a relative attenuation factor *I*(*α,β*) accounting for interferer blocking effects, obtained by normalizing the simulated molecular flux from MATLAB simulations (Section3.2, **Table 3**) to its maximum value; and (2) five empirical correction parameters (*B*_1_–*B*_5_) compensating for systematic deviations in dispersion, buoyancy, degradation, and overall amplitude. The modified model expression takes the following form Equation 58:

(58) $$c_{r}^{fit}(x,y,z,t,\alpha ,\beta )=B_{5}\frac{mI(\alpha ,\beta )}{{\left(4\pi \sqrt{B_{1}B_{2}}\right)}^{3/2}}\text{exp}(\frac{-{\left(x-ut\right)}^{2}}{4\sqrt{B_{1}B_{2}}})\text{exp }(\frac{-y^{2}}{4B_{1}})[\text{exp}(\frac{-{\left(z-\frac{B_{3}}{2}t^{2}-H\right)}^{2}}{4B_{2}})+\text{exp}(\frac{-{\left(z-\frac{B_{3}}{2}t^{2}+H\right)}^{2}}{4B_{2}})]\text{exp}(-B_{4}t)$$

where *B*_1_ and *B*_2_ are dimensionless correction factors for the lateral (*r_y_*) and vertical (*r_z_*) dispersion coefficients, respectively; *B*_3_ modifies the buoyancy-driven vertical acceleration (*PM/RT* − *ρ_b_*)*g/ρ_b_*; *B*_4_ adjusts the effective degradation rate constant *A*exp(−*E_a_/RT*); and *B*_5_ is an overall amplitude scaling factor accounting for source strength uncertainty, sensor calibration errors, and systematic deviations between the simplified model and experimental conditions. The following parameter values are used in the fitting process: emission rate *m* = 0.0016 mg, time *t* = 1 s, lateral position *y* = 0 m, vertical position *z* = 0.3 m, emitter height *H* = 0.4 m, interferer size ratio *α* = 0.5, and interferer position ratio *β* = 0.5. Nonlinear least squares fitting (Levenberg–Marquardt algorithm) is applied to determine the optimal values of *B*_1_–*B*_5_ by minimizing the discrepancy between experimental measurements and model predictions. Both experimental and theoretical curves are normalized using their respective maximum values prior to comparison. **Figure 17** illustrates the normalized comparison results under three wind speed conditions, and **Table 5** summarizes the corresponding fitting parameters and performance metrics.

**Table 5:** Fitting parameters and performance for **Figure 17**.

u	$B_{1}$	$B_{2}$	$B_{3}$	$B_{4}$	$B_{5}$	$R^{2}$	SSE	RMSE
0	0.9731	0.9413	0.9853	7.0579	0.8778	0.9983	0.005854	0.007591
0.5	0.9912	0.9721	1.0143	8.0552	0.9221	0.9972	0.007829	0.005904
1	1.0214	1.0047	1.0758	7.5994	1.2874	0.9981	0.005797	0.006984

#### 3.5.2 Impact of interferer position on concentration distribution

**Figure 18:**
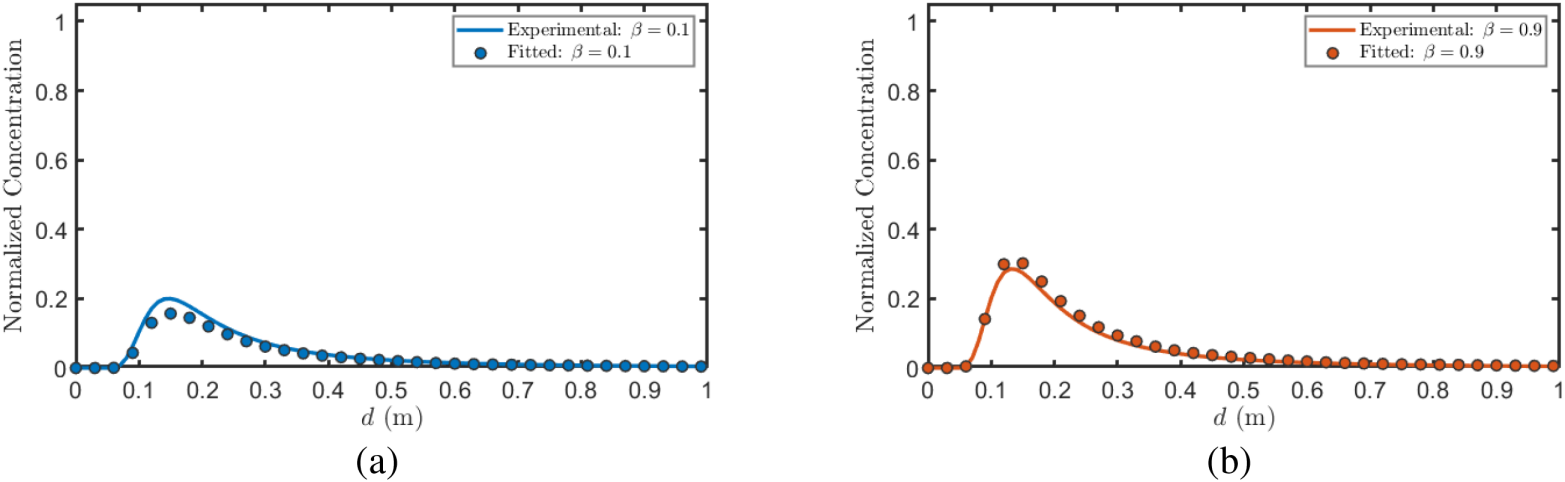
Fitting results at various interferer positions. **(a)** *β* = 0.1, **(b)** *β* = 0.9.

To systematically investigate the blocking effect of obstacles on molecular signal propagation, this section examines the concentration distribution under two interferer position configurations while keeping wind speed (*u* = 0.5 m/s), interferer size (*α* = 0.5), and other environmental parameters constant. Two position ratios are selected: *β* = 0.1 (interferer near transmitter), and *β* = 0.9 (interferer near receiver). The modified theoretical model (Equations 58) is fitted to experimental data using the same parameter correction scheme (*B*_1_–*B*_5_) and normalization procedure as described in Section 3.5.1. The relative attenuation factor *I*(*α,β*) is updated for each position configuration based on the corresponding MATLAB simulation results (**Table 3**). The corresponding normalized concentration distributions as functions of distance are presented in **Figure 18**, with the fitting parameters and performance metrics summarized in **Table 6**.

**Table 6:** itting parameters and performance for **Figure 18**.

β	$B_{1}$	$B_{2}$	$B_{3}$	$B_{4}$	$B_{5}$	$R^{2}$	SSE	RMSE
0.1	1.0568	0.9608	1.0058	0.0021	0.8891	0.9967	0.002685	0.008562
0.9	1.0275	1.0119	0.9503	0.0052	0.9653	0.9908	0.001264	0.008952

**Figure 18** presents the normalized concentration distributions under two extreme interferer placements. The near-identical concentration profiles observed at *β* = 0.1 and *β* = 0.9 can be attributed to the commutative nature of the blocking and channel attenuation processes. For *β* = 0.1 (interferer positioned near the transmitter), BVOCs experience blocking first, followed by channel attenuation; conversely, for *β* = 0.9 (interferer positioned near the receiver), BVOCs undergo channel attenuation first, then encounter the blocking effect. The final concentration distribution trends under both scenarios are in reasonable agreement with the results presented in **Figure 10**, thereby completing the preliminary validation of the theoretical framework.

## 4 Discussion

This study establishes a single-input single-output transmission model within the molecular communication theoretical framework to address plant-to-plant stress information transfer via BVOCs, with comprehensive characterization of multiple environmental factors and interference mechanisms. Unlike traditional research approaches that primarily rely on physiological indicators or compositional detection, this work adopts a communication system perspective, abstracting BVOCs emission, airborne propagation, and receiver absorption as modulation, channel propagation, and demodulation stages, respectively. While classical atmospheric dispersion models, such as the Gaussian plume model, excel at describing steady-state concentration distributions of continuous emissions over macroscopic scales, they often oversimplify the transient dynamics of discrete signaling events and lack a built-in framework for receiver absorption, signal demodulation, and information-theoretic metrics. By contrast, our molecular communication approach captures the dynamic, end-to-end information transfer process, thereby enabling quantitative description of chemical signal transmission among plants. Crucially, our ecological implications are directly grounded in our numerical and experimental evidence. Numerical simulations quantitatively demonstrate that wind speed, propagation distance, temperature, and height matching collectively determine the effective transmission range. Specifically, our data show that the received signal quantity reaches its maximum when the receiver height is approximately two-thirds of the transmitter height, providing a physically and mathematically derived explanation for vertical communication constraints often observed within real plant canopies. Furthermore, by incorporating interfering plants into the channel model and calculating their equivalent multiplicative attenuation factors, we provide an analytical, evidence-based explanation for how dense vegetation physically blocks or absorbs signaling molecules. Systematic experiments using an electronic nebulizer and an MQ-3 sensor array preliminarily validated these theoretical predictions, confirming the feasibility of stress signal detection based on OOK modulation.

Despite these theoretical and experimental advancements, translating the current model to real-world ecosystems reveals several inherent limitations. Primarily, the reliance on a SISO framework oversimplifies the multi-source BVOCs fields typical of natural plant communities. In reality, simultaneous emissions from multiple stressed plants create a complex MIMO network characterized by co-channel interference, signal overlapping, and cross-talk. While this SISO abstraction offers mathematical tractability for isolating channel dynamics, it fundamentally constrains ecological interpretability by bypassing the coexistence of numerous transmitters and receivers. Beyond this network simplification, biological reality dictates that plant stress responses involve specific blends of multiple compounds, characteristic concentration ratios, and dynamic temporal emission profiles, all of which collectively encode signaling information. Neglecting this mixture complexity inevitably reduces the model’s validity when extrapolated to natural communities. Furthermore, broader ecological complexities, such as heterogeneous canopy architectures, multi-species interactions, and background volatile noise from non-stressed vegetation, introduce additional layers of signal attenuation and misinterpretation that extend beyond our current simplified attenuation factors. From an empirical standpoint, our experimental testbed is constrained by scale limitations. Although the indoor platform successfully validates fundamental transmission mechanisms, its physical dimensions cannot realistically reproduce the complex atmospheric turbulence characteristic of natural canopies, rendering the experimental results a foundational proof-of-concept rather than a direct replication of field-scale dispersion. Ultimately, the parameter fitting process employed in our model is inherently susceptible to non-uniqueness. Because the transmission dynamics are jointly governed by multiple coupled variables, different parameter combinations could theoretically yield similar spatiotemporal concentration profiles. Although we mitigated this risk by strictly constraining our parameters within physically and biologically realistic bounds, the intricate coupling of these factors implies that fully decoupling their individual effects remains a significant mathematical and empirical challenge within the current framework.

Future work will focus on addressing these limitations to ensure that ecological implications remain strictly evidence-based. On one hand, the current framework can be extended to multi-source, multicomponent BVOCs cooperative signal models, incorporating dynamic environmental variables (e.g., humidity, solar radiation) to construct a multi-variable-driven transmission model that better reflects empirical field observations. On the other hand, the experimental platform will be continuously upgraded by introducing surrogate gases or small-molecule mixtures that more closely approximate real BVOCs behavior. In parallel, systematic experiments will be conducted at multiple distances, heights, and interference configurations using multi-point sensor arrays and adjustable wind field systems, ultimately bridging the gap toward larger-scale wind tunnel or open-field validations. Moreover, joint measurements alongside real plant physiological responses—such as stomatal conductance and fluorescence signals—will be explicitly explored to provide direct biological evidence for model parameter calibration and mechanism elucidation. Through these evidence-driven efforts, we aim to further consolidate the theoretical foundation of plant-toplant chemical communication and translate these findings into validated quantitative tools for crop health monitoring and precision agriculture.

## Data Availability

The original contributions presented in the study are included in the article/supplementary material. Further inquiries can be directed to the corresponding authors.
